# Incidence and severity of G6PI-induced arthritis are not increased in genetically distinct mouse strains upon aging

**DOI:** 10.1186/s13075-021-02596-7

**Published:** 2021-08-24

**Authors:** Nico Andreas, Sylvia Müller, Nicole Templin, Paul M. Jordan, Harald Schuhwerk, Michael Müller, Jana Gerstmeier, Laura Miek, Saskia Andreas, Oliver Werz, Thomas Kamradt

**Affiliations:** 1 Institute of Immunology, University Hospital Jena, Leutragraben 3, 07743 Jena, Germany; 2 Department of Pharmaceutical/Medicinal Chemistry, Friedrich Schiller University, Philosophenweg 14, 07743 Jena, Germany

**Keywords:** G6PI, Arthritis, Age, T cells, FLS, SPM

## Abstract

**Background:**

The incidence of rheumatoid arthritis is correlated with age. In this study, we analyzed the association of the incidence and severity of glucose-6-phosphate isomerase (G6PI)-induced arthritis with age in two different mouse strains.

**Methods:**

Young and very old mice from two different arthritis-susceptible wild-type mouse strains were analyzed after a single subcutaneous injection of G6PI *s.c*. The metabolism and the function of synoviocytes were analyzed in vitro, the production of bioactive lipid mediators by myeloid cells and synoviocytes was assessed in vitro and ex vivo by UPLC-MS-MS, and flow cytometry was used to verify age-related changes of immune cell composition and function.

**Results:**

While the severity of arthritis was independent from age, the onset was delayed in old mice. Old mice showed common signs of immune aging like thymic atrophy associated with decreased CD4^+^ effector T cell numbers. Despite its decrease, the effector T helper (Th) cell compartment in old mice was reactive and functionally intact, and their Tregs exhibited unaltered suppressive capacities. In homeostasis, macrophages and synoviocytes from old mice produced higher amounts of pro-inflammatory cyclooxygenase (COX)-derived products. However, this functional difference did not remain upon challenge in vitro nor upon arthritis reactions ex vivo.

**Conclusion:**

While old mice show a higher baseline of inflammatory functions, this does not result in increased reaction towards self-antigens in arthritis-susceptible mouse strains. Together, our data from two different mouse strains show that the susceptibility for G6PI-induced arthritis is not age-dependent.

**Supplementary Information:**

The online version contains supplementary material available at 10.1186/s13075-021-02596-7.

## Introduction

Elder people commonly exhibit a low-grade systemic, sterile inflammation, a phenomenon termed inflammaging [1]. Paradoxically, they also exhibit higher mortality to infections due to impaired immune responses, which is termed immunosenescence [2]. Although a considerable number of studies focused on altered immune mechanisms in the context of aging [3], harmonizing the partially controversial concepts of inflammaging and immunosenescence for clinical benefit still remains a challenge to research.

A prominent feature of immune aging is the age-related atrophy of the thymus. This reduces the output of naive T cells, which shifts the balance within the peripheral T cell compartment from naive to effector/memory phenotypes accompanied by a substantially reduced clonal T cell receptor (TCR) diversity [4]. An age-dependent structural alteration of the secondary lymphoid tissue architecture [5] not only hinders the migration of T cell and antigen-presenting cells (APCs) to the peripheral lymphoid organs and thereby, the costimulation of T cells by APCs [6, 7], but also disturbs the maintenance of naive Th cells in the periphery [8]. Thereby, the induction of a concerted and specific adaptive immune responses is hampered. However, an age-associated increase of low-grade inflammation can be triggered by the accumulation of misfolded proteins or improper barrier function of mucosal surfaces [9]. In addition, the defective removal of dead cells disturbs the resolution of an immune response and supports the persistence of inflammation [10]. In addition, senescent cells secreting proinflammatory mediators accumulate in various tissues and thereby increase basal inflammation [11]. This phenomenon is commonly referred to as senescence-associated secretory phenotype (SASP). SASP-induced IL-1β and TNFα drive stromal cells into senescent conditions resulting in reduced regenerative processes such as wound healing [12]. Increased basal levels of C-reactive protein (CRP) or pro-inflammatory cytokines like IL-6 or chemokines like IL-8 are generally associated with a shortened lifetime [9]. Thus, SASPs may explain a prevalent setting of inflammaging in a context of defective immune functions due to immunosenescence. How these age-related alterations in the immune system functionality are related to rheumatoid arthritis (RA) is still controversial. RA is a chronic inflammatory joint disease that often results in joint destruction and disability. A combination of risk factors including diet, microbiome composition, respiratory exposure, and genetics is thought to contribute to RA development [13]. Like RA, many autoimmune pathologies have been epidemiologically investigated for their correlation with age. Depending on the ethnicity of the investigated cohort, the incidence of systemic lupus erythematodes (SLE) increases early in life and remains constant or even decreases slowly until the age of 80 years, and subsequently declines [14]. Comparably, the incidence rate of ankylosing spondylitis sharply increases until 3rd decade of life and then slowly decreases [15]. In stark contrast, giant cell arteritis occurs at very old age [16]. In comparison to these conditions, the documented relationship of rheumatoid arthritis (RA) incidence with age appears to be enigmatic because the incidence rate peaks between the age of 55 and 85 years, but strongly declines thereafter [12, 17–19]. In contrast to the incidence, the severity of RA symptoms correlates with age and increases significantly beyond the age of 65 [20]. In line with this, the severity of bone erosions strongly correlates with the age of RA onset [21]. Fibroblast-like synoviocytes (FLS) are important for the structure of the synovial lining architecture and the production of synovial fluid constituents [22]. Upon pathological modulation, FLS are among the major drivers of the progression of RA [23, 24]. Pathologic FLS are located in the sublining layer and have originally been described as a CD90^+^ [23]. Recently, the identification has been refined to CD34^-^FAPα^+^NOTCH3^+^CD90^+^ FLS being located at the blood vessel of RA synovium, which differentiate from CD90^-^ FLS population upon activation of Notch3 signaling [23–25]. These pathogenic FLS attract and activate leukocytes to the synovial surrounding by production of large arrays of pro-inflammatory chemokines and cytokines like CCL2 and IL-6, respectively [23, 24]. However, it remains to be elucidated, whether how the homeostasis of these pathologic FLS is altered in elder individuals. Similar to the increasing incidence of RA in human beyond the age of 30, arthritis in mouse models can only be properly induced if the mouse has reached a certain age before immunization [26].

In the presented work, we used a highly synchronized model of arthritis, the G6PI-induced arthritis model [27] to investigate the relationship between onset and severity of arthritic symptoms and age. We surveyed old mice for age-related characteristics of immunosenescence and inflammaging and avoided any strain-specific particularities by inducing arthritis in DBA/1 as well as B6.NQ mice. Our data demonstrate that the immune-related changes upon organismal aging do not alter the highly synchronized onset and progression of G6PI-induced arthritis in mice.

## Methods

### Mice

B6.NQ mice express a congenic fragment containing the Aq gene from the B10.Q mice [28] and were kindly provided by Dr. Rikard Holmdahl, Karolinska Institute, Stockholm, Sweden. Terc^-/-^ mice, which exhibit a premature aging phenotype due to telomere shorting and chromosomal instability [29] were provided by Dr. Lenhard Rudolph, Fritz-Lipmann-Institute (FLI-Leibniz), Jena, Germany, and were backcrossed onto B6.NQ background. DBA/1 mice were purchased from Janvier labs, France. All mice were bred and housed in the animal facility of the University Hospital Jena. All experiments were conducted following approval by the Thüringer Landesamt für Verbraucherschutz, Bad Langensalza, Germany; registration numbers 02–041/14, 02–079/14, 02–028/15, and 02-031/15. The exact age of every mouse analyzed in this study is shown in the Supplementary Tables.

### Induction and assessment of arthritis

Recombinant human G6PI was prepared as previously described [27]. DBA/1 and B6.NQ mice were immunized by subcutaneous (*s.c.)* and intradermal (*i.d.*) injections, respectively, which consisted of 400 μg recombinant human G6PI in PBS emulsified 1:1 (vol/vol) with complete Freund’s Adjuvant (CFA; Sigma). Mice were examined for signs of arthritis at least three times per week, and the arthritis score was recorded for each mouse as described before [30]. The maximum attainable score for each mouse was 33. To summarize individual experiments, the average arthritis score was calculated in 7 day—intervals with the median days 7, 14, 21, 28, 35, 42, 49, and 56. To assess the onset of arthritis, the day 9 time point was calculated from scores at days 8, 9 and 10.

### Preparation of single cell suspensions ex vivo

All 4 limbs of the mice were collected. Upon careful removal of the skin, tendons, and muscles, the paws were separated at the wrist and ankle regions, respectively, by cutting the filaments without injuring the bones. Isolated paws were digested in 1 mg/mL collagenase type IV (*Worthington, LS004189*) in fully supplemented cell culture medium (*Dulbecco’s modified Eagle’s medium (DMEM), Sigma, D5796, high glucose);* supplemented with 10% fetal calf serum (FCS), 1 mM sodium pyruvate *(Sigma)*, 50 μM β-mercaptoethanol *(Gibco)*, 100 U/ml streptomycin/, and 10 mg/ml penicillin *(PAN Biotech)*. Tissue clumps and separated bones were removed by passing cells through cell strainers (70μm, BD Falcon), followed by a washing step in fully supplemented DMEM. Cell numbers were counted with the help of a Neubauer chamber.

The spleens and lymph nodes (LNs) were minced through a 70-μm cell strainer (BD Falcon). Splenic erythrocytes were lysed by in erythrocyte lysis buffer (0.15mM NH_4_Cl, 1mM KHCO_3_, 0.1mM Na_2_EDTA, pH 7.4) at RT for 5 min. Cells were either recovered in cell culture medium (*RPMI 1640, Sigma, R8758)*; supplemented with 10% FCS, 1 mM sodium pyruvate *(Sigma)*, 50 μM β-mercaptoethanol *(Gibco)*, 100 U/ml streptomycin/, and 10 mg/ml penicillin *(PAN Biotech)* for later cell culture experiments (= fully supplemented), or in PBA-E *(PBS, 5 mg/mL, BSA, 10 mM NaN*_*3*_*, 2 mM EDTA)* for flow cytometrical cell analysis.

### Synoviocyte cell culture

Upon purification, synoviocytes were seeded fully supplemented DMEM in a T75 culture flask and incubated at 37°C and 5% CO_2_ for 3 days. After 3 days, the non-adherent cells were removed by replacement with fresh medium. At 90% confluence the cells were detached by trypsinization (0.25% Trypsin in serum-free DMEM) for 5 min at 37°C, and recovered cells were passaged 1:3 to a new T75 culture flask.

### Antigen-specific Th cell stimulation

To investigate the reactivity of Th cells, single cell suspensions of pooled lymph nodes (inguinal, brachial, axillary) were prepared and 10^7^ cells per mouse were restimulated. To analyze the functional capacities of Th cells, cell suspensions were restimulated with aCD3/aCD28 beads (Dynabeads Mouse T-Activator CD3/CD28, Gibco) in a ratio of 1:2 (beads:cells). To analyze G6PI-specific Th cells, 5 x 10^6^ single cells were restimulated with 100 μg G6PI in 500 μl. After 2h of stimulation, Brefeldin A (Sigma) was added to the preparation for further 4h. Subsequently, the cells were fixed with 2% formaldehyde/PBS for intracellular cytokine staining.

### Flow cytometry

For flow-cytometric analysis, cell suspensions (synoviocytes: 500,000 cells/sample; splenocytes or lymph node cells: 5 x 10^6^ cells/ sample) were incubated in 2 μg/mL polyclonal rat IgGs (Jackson Immunoresearch laboratories) for 10 min at 4°C and subsequently stained with 2 μg/mL of fluorophore-conjugated antibodies directed against the surface antigens CD45 (clone REA737, VioGreen, Miltenyi), CD90.2 (APC-eFluor780, clone 53-2.1, Invitrogen), F4/80 (clone BM8, PE/Cy7 or APC/Cy7, BioLegend), CD11b (AlexaFluor700, clone M1/70, eBioscience), CD11b (BV605, clone M1/70, BioLegend), Ly6G (clone 1A8, AlexaFluor700, BioLegend), SiglecF (clone REA798, APC, Miltenyi), or MHC-II (clone M5/114.15.2, PE, eBioscience), CD4 (clone GK1.5, APC/Cy7, BioLegend), CD8 (clone 53-6.7, PE/Cy7, BioLegend), CD25 (clone 3C7, APC, BioLegend), CD62L (clone MEL-14, FITC, BioLegend), CD44 (clone REA664, PE/Cy7, Miltenyi), CD11c (clone N418, PE, Invitrogen), and CD19 (clone 6D5, AlexaFluor700, BioLegend) as indicated. Before analysis, 4′,6-Diamidin-2-phenylindol (DAPI, *Sigma)* or Propidium iodide (PI, *Miltenyi)* was added to the samples. Samples were analyzed using a BD LSRII flow cytometer and FlowJo software (TreeStar, Inc.).

The formaldehyde-fixed cells were incubated in 1 μg/mL polyclonal rat IgG (Jackson Immunoresearch laboratories) in PBA-E with 0.5% (w/V) saponin for 10 min at 4°C and subsequently stained with fluorophore-conjugated antibodies directed against CD4 (clone GK1.5, AlexaFluor700, BioLegend), CD154 (clone MR1, PE, Invitrogen), IFNγ (clone XMG1.2, APC/Cy7, BioLegend), IL-4 (clone 11B11, BV421, BioLegend), IL-17A (clone TC11-18H10.1, APC, BioLegend), GM-CSF (clone MP1-22E9, PerCP/Cy5.5, BioLegend), and TNFα (clone MP6-XT22, BV510, BioLegend) in PBA-E with 0.5 % (w/V) saponin in darkness at 4°C for 20 min. Upon washing with PBA-E with 0.5 % (w/V) saponin, the samples were recovered in PBA-E for flowcytometric analysis.

For transcription factor staining, the FoxP3 staining kit (Invitrogen) was used according to the manufacturer’s protocol. In brief, cells were fixed with the Fix/Perm buffer at 4°C for 60 min, followed by washing with permeabilization buffer and staining with antibodies against CD4 (clone GK1.5, APC/Cy7, BioLegend), FoxP3 (clone MF14, BV421, BioLegend), RORγt (clone Q31-378, PE, BD Bioscience), GATA3 (clone 16E10A23, APC, BioLegend), and Helios (clone 22F6, PerCP-eFluor710, Invitrogen) in permeabilization buffer at 4°C for 45 min. Subsequently, the samples were washed with permeabilization buffer and resuspended in PBA-E for flowcytometrical analyses.

### Suppression assay

Living CD11c^high^ cDCs, CD4^+^CD25^+^ Tregs and CD4^+^CD25^-^CD62L^+^CD44^-^ naive Th cells were isolated from splenocytes with a FACS ARIA III sorter (BD). 10^7^ naive Th cells/mL were stained with 5 μM pre-activated CFDA-SE in PBS/0.5% BSA and incubated for 5 min. Upon washing with PBS/0.5% BSA, 10^5^ CFSE-labeled Th cells/well were cultured in the presence of 1 μg/mL of soluble α-CD3ε and 2x10^4^ cDCs in a 96-well plate for 3 days. Th cells were composed of Tregs and naive Th cells in the indicated ratios. CFSE dilution was analyzed among CD4^+^CD11c-CFSE^+^ culture cells, and the proliferation index represents the total number of recovered cells divided by the number of starting cells calculated by the amount of CFSE dilution cycles of each cell.

### Analysis of lipid mediators (LM)

Cells obtained from the peritoneal lavage was cultured in RPMI 1640 supplemented with 10% FCS, 2 mmol/L glutamine (Biochrom/Merck, Berlin, Germany), and penicillin-streptomycin (Biochrom/Merck) for 24 h and washed once with PBS. Peritoneal lavage cells were stimulated in PBS containing 1mM CaCl_2_ with 1% *Staphylococcus (S.) aureus* conditioned medium (SACM) of the 6850 wt strain or 1% brain heart infusion as vehicle for 180 min at 37°C. Preparation of SACM is described by Jordan et al. [31]. Synovial cells of the small joints were isolated and directly stimulated in PBS containing 1 mM CaCl_2_ with 2.5 μM Ca^2+^-ionophore A23187 (Cayman Chemical/Biomol GmbH, Hamburg, Germany) or vehicle for 180 min. Cells were transferred into ice-cold methanol containing 10 μL of deuterium-labeled internal standards (200 nM d8-5S-HETE, d4-LTB_4_, d5-LXA_4_, d5-RvD2, d4-PGE_2_, and 10 μM d8-AA) to facilitate quantification and sample recovery. Sample preparation was adapted from previously published studies [32]. In brief, samples were kept at −20°C for 60 min to allow protein precipitation and transferred into acidified H_2_O followed by solid phase extraction (Sep-Pak® Vac 6cc 500 mg/ 6 mL C18; Waters, Milford, MA).Upon drying by evaporation (TurboVap LV, Biotage, Uppsala, Sweden), lipid mediator (LM) profiling was performed with an Acquity™ UPLC system (Waters, Milford, MA, USA) and a QTRAP 5500 Mass Spectrometer (ABSciex, Darmstadt, Germany) equipped with a Turbo V™ Source and electrospray ionization as previously published [33, 34]. The following groups of LM were analyzed: specialized pro-resolving mediators (SPM, represents the sum of PD1, PDX, RvD2, RvD5, MaR1, MaR2, and LXA_4_), COX products (represents the sum of PGE_2_, PGD_2_, PGF_2α_, and TXB_2_), 5-LOX products (represents the sum of LTB4, trans-LTB4, and 5-HETE), 15-LOX products (represents the sum of 17-HDHA, 15-HEPE, and 15-HETE), and 12-LOX products (represents the sum of 14-HDHA, 12-HEPE, and 12-HETE).

### Metabolic assay

Oxygen consumption rate (OCR) and Extra Cellular Acidification Rate (ECAR) were measured using the Agilent Seahorse XFp Cell Energy Phenotype test kit at the XFe96 Extracellular Flux Analyzer (Agilent) according to manufacturer’s instructions. FLS were seeded onto XF96 culture microplates (5000 cells per well) in complete FLS medium and allowed to adhere and grow for 24 h in a 37°C humidified incubator with 5% CO_2_. Measurements of metabolism were performed in unbuffered media. One hour prior to the start of the assay, the media were replaced by unbuffered (pH 7.4) DMEM containing 2 mM glutamine, 1 mM sodium pyruvate,10mM glucose, and incubated at 37°C without CO_2_. The results were normalized to cell numbers by photometric measurement of Bradford staining of the cultured cells after the metabolic assay.

### Invasion assay

Transwell cell culture inserts (pore size 8 μm, Corning) were coated with MEM (Sigma) containing 40% collagen (PureCol, Sigma) at 37°C for 90 min. 2×10^4^ FLS were seeded into the precoated insert in serum-free DMEM and introduced into a 24-well plate containing fully supplemented DMEM. After 36 h, the inserts were washed in PBS and subsequently incubated in crystal violet solution (Sigma) at RT in the dark for 20 min. Upon repeated washing and subsequent drying, the membranes were removed from the inserts and coated with Entellan solution (Millipore) on microscope slides. Pictures of whole membranes were obtained by binning in a 10x magnification with a Zeiss Axioobserver Z1. The ratio of the stained surfaces was calculated with the Biovoxxel Toolbox in the Fiji/ImageJ software.

### Senescence assay

FLS of minimum P3 were cultured until 90% confluency. Upon removing culture medium by repeated washing with PBS, cells were fixed with 2% formaldehyde/H_2_O at RT for 15 min. Upon repeated washing, fixed cells were stained with a Senescence β-Galactosidase Staining Kit (Cell Signaling Technology #9860) according to manufacturer’s protocol.

### Statistics

All data were tested for normal distribution using Shapiro-Wilk test. Equal variance was tested using Brown-Forsythe test. If data were normally distributed with equal variance, a two-sided Student’s *t* test was performed. Otherwise, Mann-Whitney rank sum test was performed. Statistical significance is indicated as ^***n.s.***^non-significant; *p<0.05, **p<0.01, and ***p<0.001.

## Results

**Fig. 1:**
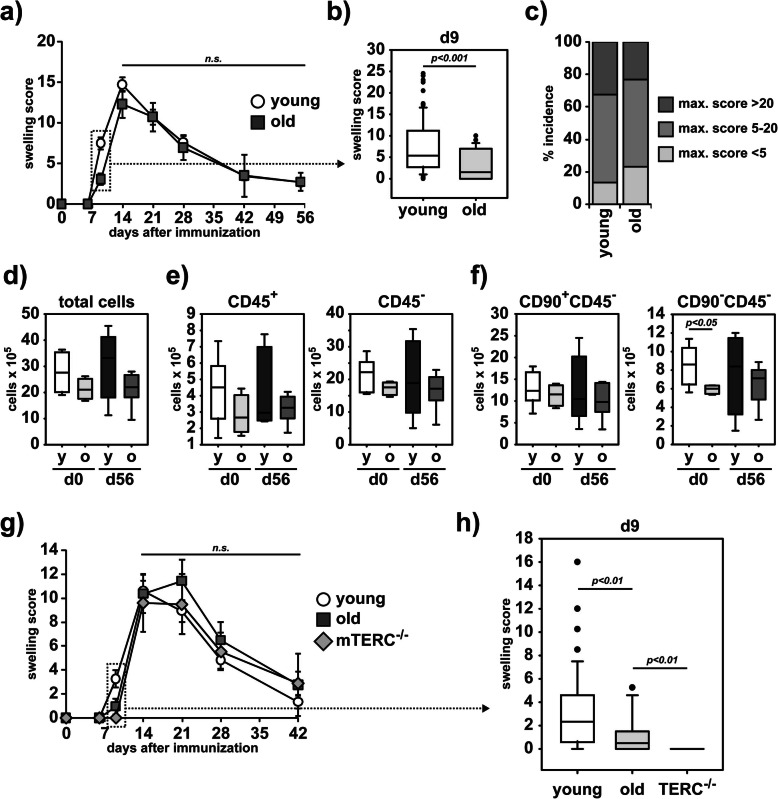
Young and old mice are similarly susceptible to G6PI-induced arthritis. Young (average age of 10 weeks, *n*=74) or old (average age of 96 weeks, *n*=26) DBA/1 mice were immunized with G6PI. Arthritis severity was scored after immunization. **a** The scores were summarized in the diagram in intervals with the average time point indicated on the *x*-axis. **b** Score at day 9 is shown separately. **c** Percentage of mice developing arthritis of low severity (max. score < 5), intermediate severity (max. score of 5–20) or strong severity (max. score > 20). **d–f** Before induction and after recovery from arthritis, synovial cells of the small joints were extracted and total number of all cells recovered (**d**), CD45^+^ cells or CD45^-^ cells (**e**), and CD90^+^CD45^-^ or CD90^-^CD45^-^ cells were determined (**f**). Individually analyzed mice were grouped into young d0 (average age of 16 weeks, *n*=8), young d56 (average age of 25 weeks, *n*=5), old d0 (average age of 66 weeks, *n*=4), or old d56 (average age of 89 weeks, *n*=6). **g** Young B6.NQ (open circles, average age of 10 weeks, *n*=49), generation 4 B6.NQ/Terc^-/-^ (gray diamonds, average age of 40 weeks, *n*=16), or old B6.NQ (dark gray squares, average age of 70 weeks, *n*=11) mice were immunized with G6PI in CFA *s.c.*. Arthritis was scored for 42 days. **h** Score of the day 9 interval is separately shown as box plot (n.d. = not detectable). Statistical testing was performed as described in the methods section. n.s. non-significant

**Fig. 2:**
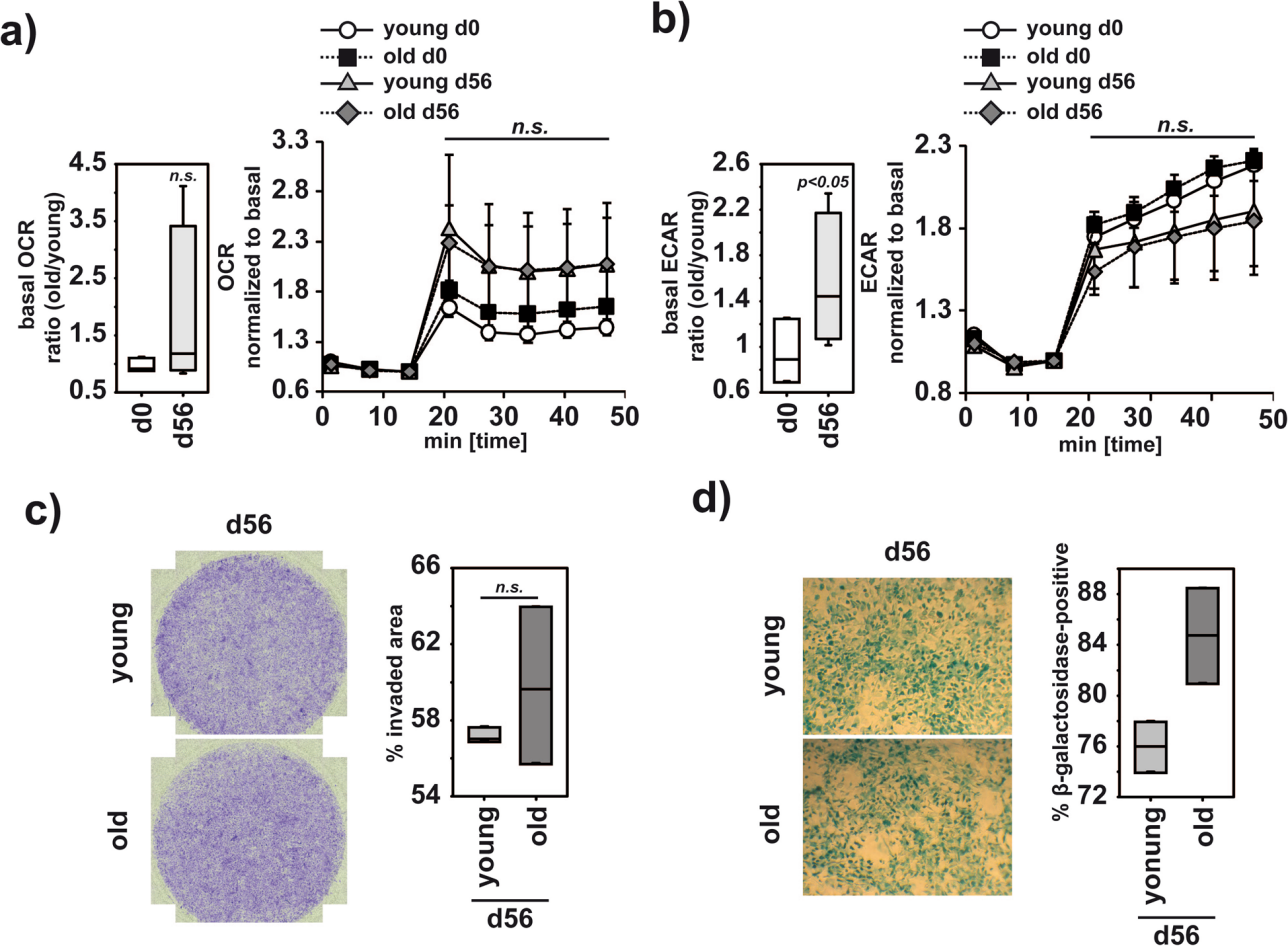
Upon arthritis FLS from old mice are more senescent and show higher glycolysis. Young and old mice were immunized with G6PI. Before or 56 days after immunization, synovial cells were extracted from the small joints and cultured. **a, b** At passage P4 FLS metabolism was analyzed. Basal OCR (**a** left) or ECAR (**b** left) were calculated as ratio of the values obtained for FLS from old mice vs FLS from young mice at d0 (average age: young: 13 weeks, *n*=3, old: 108 weeks, *n*=3) or d56 (average age young: 32 weeks, *n*=4, old: 89 weeks, *n*=4). Metabolic curves were normalized to the basal OCR (**a** right) or basal ECAR (**b** right) before addition of FCCP and oligomycin (1 μM each), and the induced maximal metabolic capacities are summarized in the diagrams. **c** FLS extracted 56 days after immunization were tested at P4 in a collagen matrix invasion assay. After 36 h, the numbers of migrated cells were analyzed. Representative results are shown in the left panel. Experiments are summarized in the diagram (average age: young: 16 weeks, old: 96 weeks, *n*=3). **d** At passage P4, FLS extracted 56 days after immunization were tested in a senescence assay and the percentage of senescent cells (defined as βGalactosidase-positive) was analyzed. Representative results are shown in the left panel. Data are summarized in the diagram (average age: young: 16 weeks, old: 95 weeks, *n*=2). Statistical testing was performed as described in the “Methods” section. n.s. non-significant

We induced arthritis in young (average 13 weeks) and very old (average 93 weeks) DBA/1 mice by injection of G6PI/CFA. The synchronized incidence and progression of arthritis allow us to validate age-dependent severity, onset, and susceptibility in genetically identical inbred mice. Surprisingly, there was no significant difference in the incidence or severity of arthritis between young and old mice (Fig. 1a). However, the onset of arthritis was slightly delayed in old mice (Fig. 1b), predominantly in old male mice (Supplement-Figure 1 a-c). The percentages of mice developing mild and severe symptoms were similar in young and old cohorts (Fig. 1c). When we recovered cells from the small joints of young and old mice before or after the arthritis induction, we yielded similar numbers of synoviocytes (Fig. 1d). The numbers of CD45^+^ hematopoietic cells and CD45^-^ stromal cells were also similar in both groups at d0 (Fig. 1e). These similarities were supported on histological levels by an intact bone structure (Supplement-Figure 2 a) and by the complete recovery from any joint infiltration (Supplement-Figure 2 b) beyond day 56 after immunization with G6PI in young and old mice. While we detected no alteration of the overall CD90^+^CD45^-^ FLS population between young and old mice at any time point investigated, we recovered less CD90^-^CD45^-^ FLS from the small joints of non-immunized old mice compared to young mice (Fig. 1f). Although we did not focus on FLS subpopulations in detail, that overall difference of CD90^-^CD45^-^ FLS between young and old mice observed in naïve mice was no longer detectable at day 56 after arthritis induction (Fig. 1f).

The genetic background of mice is known to be a factor for the bias of immune responses, e.g., with C57BL/6 mice and BALB/c mice being prone to Th1 or Th2-mediated responses, respectively [35]. To exclude strain-dependent differences in an age-associated susceptibility to arthritis, we investigated a G6PI-induced arthritis in young and old B6.NQ mice [28]. In addition, we used an established genetic mouse model for premature aging, the Terc^-/-^ mouse, which rapidly ages when bred *inter se* for 4 generations [29]. Strikingly, B6.NQ and B6.NQ/Terc^-/-^ mice fully confirmed our previous data using DBA/1 mice with regard both to the similar clinical course of the G6PI-induced arthritis in young and old mice (Fig. 1g) and the delayed onset of arthritis in old mice (Fig. 1h). In contrast to DBA/1 mice, in which female mice did not display any delay in the onset of arthritis, female old C57BL/6 mice did show a delayed onset (Supplement-Figure 1 d, e).

### After recovery from arthritis, synovial fibroblasts display increased signs of senescence in old mice

In RA, FLS have been reported to be major drivers of disease progression and are characterized by an increased aggressiveness with enhanced invasive functions leading to massive joint destruction [23, 24]. Alterations in metabolic pathways were reported to regulate such pathologic mechanisms and thereby impact the progression of arthritis [36]. When we tested the metabolic activity of FLS from young and old mice, we did not detect significant differences in the basic oxygen consumption rate (OCR) (Fig. 2a) or in the extra cellular acidification rate (ECAR), which represents a measure for glycolytic activity (Fig. 2b). Differences in the oxygen consumption between young and old FLS upon recovery from arthritis did not reach statistical significance (Fig. 2a). In contrast, we detected a statistically significantly increased glycolytic activity in FLS from old mice 56 days after G6PI-immunization (Fig. 2b). However, upon challenge, the maximal capacities to consume oxygen or to perform glycolysis were similar in young and old animals (Fig. 2a, b). To test whether an increased glycolytic activity is associated with an invasive, pathologic fibroblast activation as described in rheumatoid arthritis [37], we performed a collagen-based invasion assay to test FLS from young and old mice obtained at day 56 after arthritis induction after the mice had recovered from arthritis. Invasion capacity was not significantly increased in FLS from old mice compared to FLS from young mice, and thus, the observed basal metabolic differences did not translate into functional differences (Fig. 2c). This was supported by the absence of destruction and infiltration of the small joints (Supplement-Figure 2). Interestingly β-galactosidase, which is a commonly used marker for senescent cells [38] and increases upon continuous replication [39], was increased in FLS from old mice (Fig. 2d).

### Basal differences in inflammatory mediator production disappear upon arthritis resolution

**Fig. 3:**
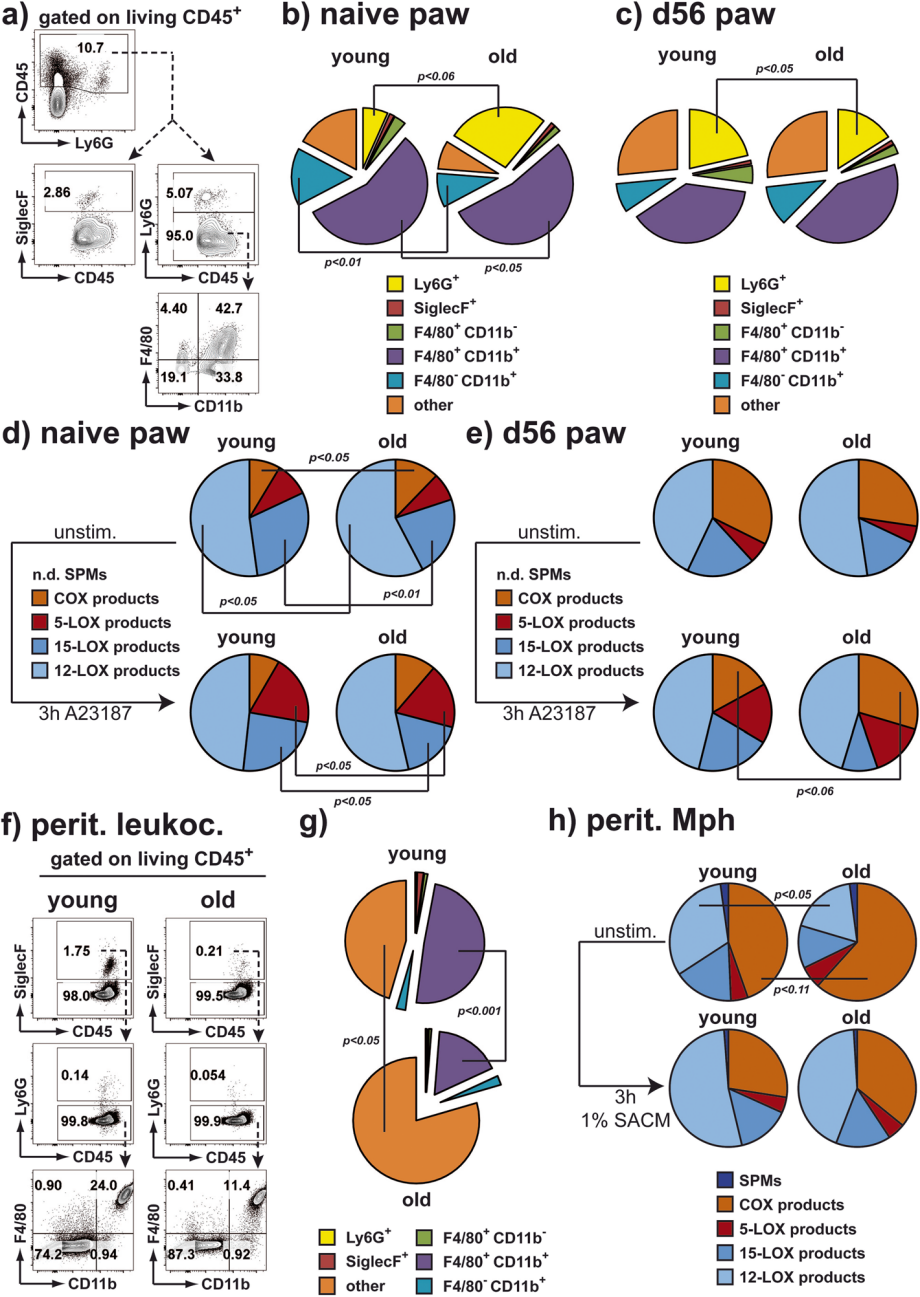
Enhanced immune effector cells and function in old mice are normalized upon challenge. **a**–**c** Synoviocytes from the small joints were extracted, and their composition was analyzed by flow cytometry. Gating strategy for myeloid subsets is shown in **a**. Pie charts show the frequencies of Ly6G^+^ neutrophils (*n*=6–12 mice), SiglecF^+^ eosinophils (*n*=2–8 mice/group), F4/80^+^CD11b^-^ (*n*=2–5 mice/group), F4/80^+^CD11b^+^ (*n*=2–5 mice/group), F4/80^-^CD11b^+^ (*n*=2–5 mice/group), and Ly6G^-^SiglecF^-^F4/80^-^CD11b^-^ (other; *n*=2–5 mice/group) subsets among synoviocytes of young (average age of 14 weeks) and old (average age of 85 weeks) mice before (**b**), or of young (average age of 22 weeks) and old (max. *n* = 8 mice, average age of 97 weeks) mice 56 days after (**c**) immunization with G6PI. **d**, **e** Synoviocytes from the small joints were extracted and directly stimulated with 2.5 μM of the Ca^2+^ ionophore A23187 or vehicle (unstim) for 3 h. Subsequently, lipid mediators were quantified by UPLC-MS-MS. Amounts of lipid mediators were related to the amounts of free fatty acids, and frequencies of metabolites among all analyzed mediators are depicted in the pie charts representing synovial cells of young (average age of 9 weeks) and old (average age of 102 weeks) mice before (**d**, *n*=3 mice/group); or of young (average age of 29 weeks) and old (average age of 102 weeks) mice 8 weeks after G6PI immunization (**e**, *n*=6 mice/group). **f, g** Peritoneal lavage cells from young or old mice were analyzed by flow cytometry. Representative plots are shown in (**f**). Pie charts (**g**) show summarized lavage compositions from young (left, *n*=11 mice, average age of 10 weeks) or old (right, *n*=11 mice, average age of 106 weeks). **h** Peritoneal lavage cells from young (*n*=7 mice in 4 analyses, average age of 16 weeks) and old mice (*n*=9 mice in 4 analyses, average age of 86 weeks) were cultured overnight to recover peritoneal macrophages. Subsequently, adherent cells were stimulated with 1% SACM or vehicle (unstim) for 3 h or left untreated. Lipid mediators were quantified by UPLC-MS-MS. Amounts of lipid mediators were related to the amounts of free fatty acids and frequencies of metabolites among all analyzed mediators are depicted in the pie charts (*n*=4 mice/group). All pie charts show average values. Statistical testing was performed as described in the methods section

Flow cytometric analyses of synovial cells (Fig. 3a) revealed an increased frequency of neutrophils in old mice in steady-state conditions (Fig. 3b). In contrast to this, the frequencies of both, tissue macrophages (F4/80^+^) and proinflammatory monocytes (F4/80^-^) among CD11b^+^ myeloid cells were reduced in old mice (Fig. 3b). Upon recovery of young mice from arthritis, the neutrophil frequencies were higher than before arthritis induction and even exceeded the frequencies in old mice (Fig. 3b, c). Lipid mediators regulate inflammation, while COX-derived prostaglandins (PG) and 5-lipoxygenase (LOX)-derived Leukotrienes (LT) support inflammatory processes, and 12/15-LOX-derived specialized proresolving mediators (SPM) promote resolution of inflammation and tissue repair [40]. In line with the increased frequencies of neutrophils in the joints of old mice under steady state conditions, we detected higher levels of proinflammatory COX products in the small joints of non-immunized old mice compared to young mice (Fig. 3d). This enhanced production of COX products as well as an elevated generation of 12-LOX products by synoviocytes from old mice was no longer statistically significant upon activation with the pro-inflammatory stimulus Ca^2+^-ionophore A23187 (Fig. 3d). Notably, the 15-LOX products were produced at slightly lower levels by synoviocytes from old mice during homeostasis, which was not altered upon stimulation with A23187 (Fig. 3d). While the levels of 5-LOX products produced by synoviocytes in steady state were comparable, their induction by A23187 stimulation was slightly lower in synoviocytes from old mice (Fig. 3d). Upon arthritis, the lipid mediator profiles were similar between synoviocytes from young or old mice (Fig. 3e). However, we could detect a slight increase in the capacity to produce pro-inflammatory COX products in synoviocytes from old mice upon arthritis when stimulated with A23187 (Fig. 3e). Within tissues, macrophages are important for tissue homeostasis via their production of lipid mediators and the removal of apoptotic or dead cells [41]. Considering that macrophages are major constituents of the protective barrier around the joint space [42] and are highly sensitive to changes in the tissue environment throughout the body, e.g., by detecting pathogenic stimuli upon infection [43], we analyzed whether we could detect similar age-related changes in the peritoneum. Reflecting the alteration of the cellular composition in the small joints of old mice, we detected decreased frequencies of F4/80^+^CD11b^+^ macrophages in the peritoneal cavity in old mice compared to young mice (Fig. 3f, g). In line with earlier reports, [44] we detected decreased frequencies of myeloid cells in the peritoneal lavage fluid of old mice and increased frequencies of B and T lymphocytes (Supplement-Figure 3). Comparable to synoviocytes, macrophages from old mice produced larger amounts of proinflammatory COX products under homeostatic conditions (Fig. 3h). However, upon challenge of these macrophages with SACM to activate all inflammation-related pathways [31], the age-related functional differences disappeared (Fig. 3h). Collectively, while the impact of age was clearly visible in steady state represented by increased production of pro-inflammatory lipid mediators and accumulation of neutrophils in the joints of old mice, the capacities to react to proinflammatory stimuli and to attract neutrophils remained comparable between young and old mice.

### Reduced T helper lymphocytes display an increased autoimmune character

**Fig. 4:**
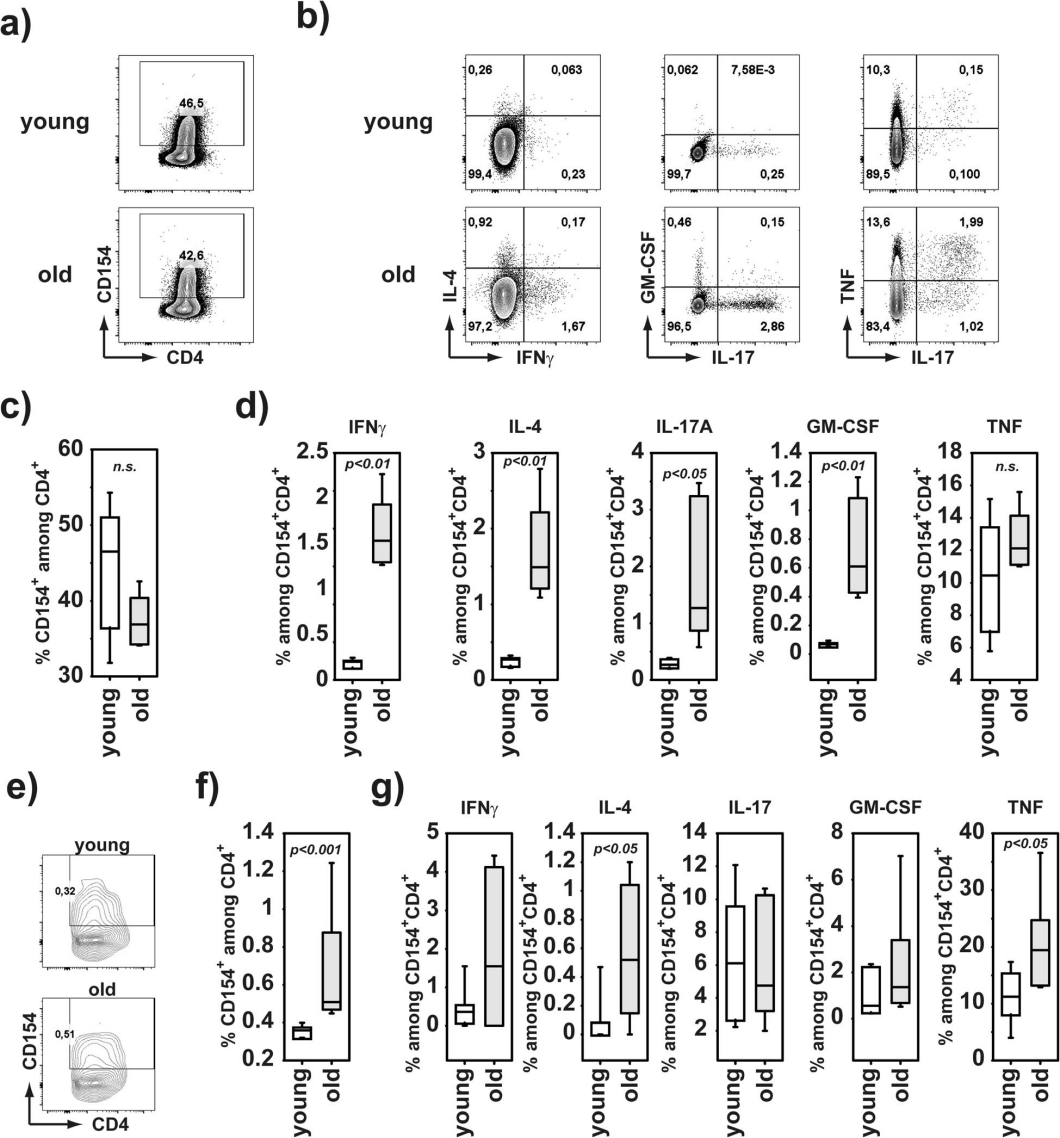
Old mice generate more TNFα-producing G6PI-specific Th cells. **a**–**d** Peripheral LN cells from young (average age of 8 weeks, *n*=5) or old (average age of 103 weeks, *n*=5) mice were restimulated with anti-CD3/CD28 beads (0.5 beads/cell) for 6h, fixed and intracellularly stained for IFNγ, IL-4, IL-17A, GM-CSF, and TNFα in combination with CD154 and CD4. FACS plots show representative staining for CD154^+^ among CD4^+^ cells (**a**) and for cytokine producers among CD154^+^CD4^+^ cells (**b**). Diagrams show average frequencies of CD154^+^ cells among CD4^+^ cells (**c**) or of cytokine producers among CD154^+^CD4^+^ cells (**d**). **e–g** Peripheral LNs were collected from young (average age of 9 weeks, *n*=7) and old mice (average age of 89 weeks, *n*=7) at day 6 after immunization with G6PI. Whole LN cells were restimulated with G6PI for 6h, fixed and stained intracellularly for cytokines in combination with CD154 and CD4. FACS plots show representative staining for CD154^+^ among CD4^+^ cells (**e**). Diagrams show average frequencies of CD154^+^ among CD4^+^ cells (**f**) or of indicated cytokines among CD154^+^CD4^+^ (**g**). Statistical testing was performed as described in the “Methods” section. n.s. non-significant

Despite the reduced overall cellularity of spleen, thymus and peripheral lymph nodes (periLNs) in old mice (Supplement-Figure 4 a), the frequency of CD4^+^ but not CD8^+^ single positive Th cells among the CD45^+^ thymocytes was increased when compared to young mice (Supplement-Figure 4 b). This was exclusively attributable to the Foxp3^-^ CD4^+^ Th cells (Supplement-Figure 4 c). The increased frequency of FoxP3^-^CD4^+^ Th cells in the thymus of old animals was not reflected in the spleen (Supplement-Figure 4 d). Immigration of lymphocytes to periLNs is impaired in old individuals due to distorted architecture [5]. In line with that we observed a massive decrease of FoxP3^-^ CD4^+^ Th cells among the CD45^+^ cells in the periLNs (Supplement-Figure 4 e). We detected unaltered GATA3^+^ or RORγt^+^ FoxP3^-^ Th cells among hematopoietic cells (Supplement-Figure 4 f, g), which indicated that this drop in FoxP3^-^ Th cells was not due to reduced Th effector/memory cells. Helios, which is a transcription factor strongly associated with Treg characteristics [45] was increased in FoxP3^-^CD4^+^ Th cells from old mice (Supplement-Figure 4 f, g). Helios expression in nonTreg cells is associated with a Treg-alike state [46], which suggested that the pool of effector/memory Th cells in old mice was efficiently constrained, while the newly generated CD4^+^ Th cells decreased in old mice compared to young mice. Alongside the fully functional capacity of myeloid cells to produce lipid mediators upon activation (Fig. 3), young and old T cells responded similarly to TCR stimulation via CD3/CD28 (Fig. 4 a, c). However, activated CD154^+^ Th cells in the draining lymph nodes of old mice produced more IFNγ, IL-4, IL-17A, and GM-CSF, but not TNFα, than those of young mice (Fig. 4b, d). Upon immunization with G6PI, we detected an enhanced frequency of G6PI-specific Th cells in the pLN of old mice compared to young mice (Fig. 4e, f). Among these G6PI-specific Th cells, we observed significantly higher frequencies of TNFα producing Th cells, whereas the frequencies of IFNγ, IL-4, IL-17A, or GM-CSF producers remained similar among the G6PI-reactive Th cells from old and young mice (Fig. 4g). Collectively and in line with former reports [4], we observed a preservation of peripheral Th effector/memory in arthritis-susceptible old mice. However, we found more TNFα-producing autoreactive G6PI-specific Th cells upon immunization of old mice.

### Tregs functionality is not associated with aging

**Fig. 5:**
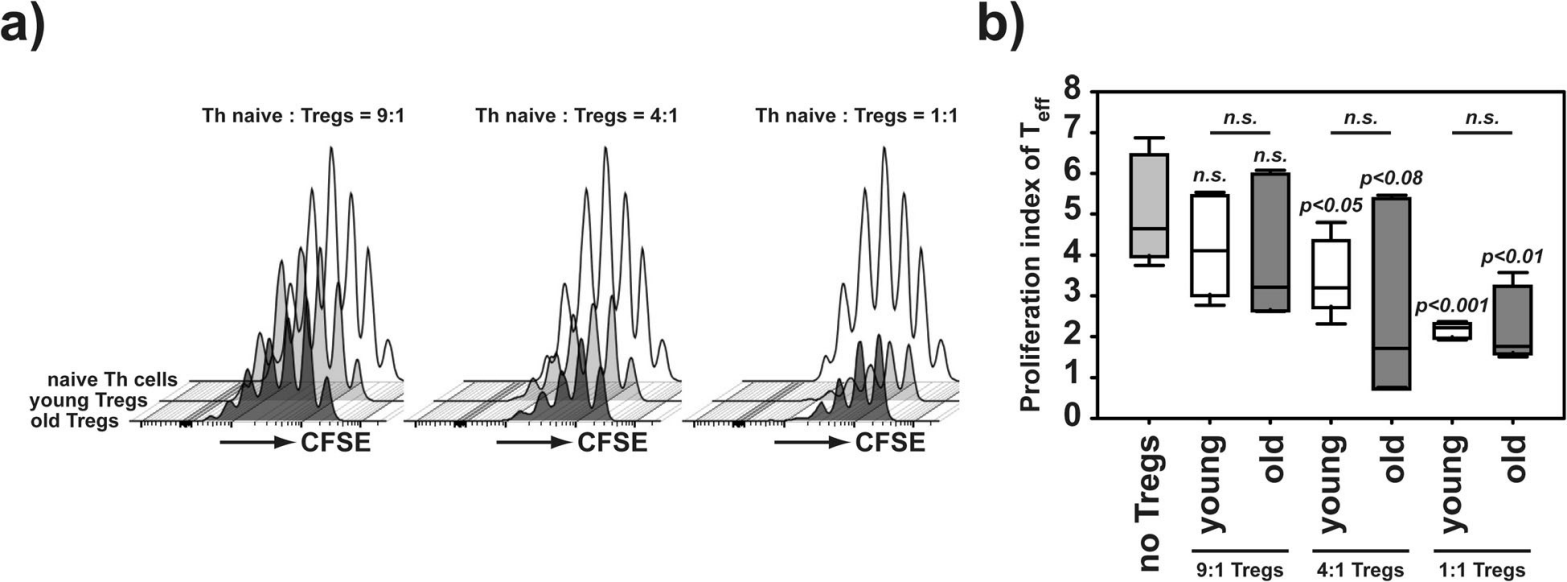
Tregs from young and old mice have similar suppressive capacities. **a**, **b** In 3 independent experiments, CD25^+^CD4^+^ regulatory T cells were sorted from young (average age <12 weeks, *n*=8) or old (average age >81 weeks, *n*=9) mice. Tregs were cocultured with CFSE-labeled CD25^-^CD62L^+^CD44^-^CD4^+^ cells from young mice in the presence of splenic conventional DCs from young mice and soluble anti-CD3ε antibodies for 3 days. CFSE dilution was analyzed (**a**), and the proliferation index was calculated as a measure representing the proliferation cycles/starting cell. Different ratios of Tregs and Th cells were analyzed as shown in the diagrams (**b**). The “noTreg” control is identical in all ratio combination of Tregs-Th cell ratios displayed. Statistical testing was performed as described in the methods section. n.s. non-significant

We were intrigued by the controversial finding that more pathogenic Th were induced in old than in young mice, without aggravating the peak response or resolution of the G6PI arthritis. We previously demonstrated that Tregs suppress autoreactive Th cells to maintain the immune balance and are required to limit severity of arthritis, eventually preventing chronicity in the G6PI arthritis model [47]. To analyze, whether an enhanced Treg functionality compensating for the systemic low-grade inflammation in old G6PI-susceptible mouse strains ultimately resulted in an unaltered arthritis response, we performed suppression assays using Tregs from young and old mice to suppress proliferation of naïve Th cells from young mice. Surprisingly, we did not observe any change in the ability of Tregs from old mice compared to their young counterparts to suppress Th cell proliferation (Fig. 5a, b). In summary, the suppressive ability of Tregs is not altered in old mice. Therefore, we conclude that the enhanced relative number of Tregs (Supplement-Figure 4 e) outbalances the enhanced numbers of G6PI-reactive Th cells in old mice.

## Discussion

In contrast to rheumatic disorders with an onset early in life like SLE [14, 17] and ankylosing spondylitis [15], or with an onset very late in life like giant cell arteritis [16], the correlation of RA to aging appears to be enigmatic [12, 17]. Despite the strong increase of RA incidence up to the 5th decade, beyond the age of 55, the incidence rate slope turns to flatten out and it even drops in the 8th decade [12, 17–19]. While the pathology of RA has been transferred to animal models to investigate mechanisms of arthritis by immunization of susceptible strains, by generation of genetic models or by transfer of serum containing arthritogenic antibodies, the correlation of age, and the progression of arthritis has not been studied, yet. To address this observation experimentally, we used the G6PI-induced arthritis model in DBA/1 and in a susceptible BL/6 mouse line, the B6.NQ mouse line [28]. The G6PI-induced arthritis model is predestined to age-related arthritis experiments due to its synchronicity of onset and recovery phase [27]. Genetically induced, spontaneous arthritis models such as SKG [48] or TNFα-tg [49] non-synchronously develop only at a certain age and thus cannot be used to address an arthritis-age-relationship. Passive models of arthritis induction like CAIA [50] or K/BxN-STIA [51] display synchronous onsets, but are not applicable to investigate age-related mechanisms of arthritis, because they circumvent the activation of T cell compartment, which is affected by age-related immunosenescence [52]. While the commonly used arthritis model of collagen-II-induced arthritis (CIA) involves T cell activation, it displays considerable variability of incidence and synchronicity [53, 54]. Employing the highly synchronous G6PI-induced arthritis mouse model in the present study, we did not observe a different arthritis progression correlating with age. The major period of investigation is between day 9 (onset) and day 14 (maximum), and therefore, the initial age of the young mice selected is not majorly changed by the length of the experiment. The use of DBA/1 as well as C57BL/6 background excluded a strain-specific phenomenon that was further supported by the fact that premature-aged B6.NQ/Terc^-/-^ mice [29] developed a similar arthritis progression. In a recent 8-year follow-up study in a large cohort, the disease course upon onset of RA was independent from sex and age [55], which fits our results. Interestingly, we observed a slight delay in the onset of arthritis in old mice (Fig. 1a, b). In DBA/1 mice that delay did not affect female mice and was predominantly associated with old male mice (Supplement-Figure 1 a-c). Surprisingly, old female C57BL/6 mice did show a delayed development of G6PI-induced arthritis comparable to old male DBA/1 mice (Supplement-Figure 1 d, e). Thus, a sex-specific age-related difference in the strength of arthritis is either strain-dependent, or the cohort we investigated for sex-specific discrimination of the age-related changes was too small. However, the investigation of sex-specific age-related alterations is beyond the focus of the presented study, but should be taken into consideration in future investigations. Of note, men younger than 40 years of age have the least disease activity and pain, while females older than 70 years show the worst disease characteristics [55]. In contrast to the generally similar arthritis responses of young and old mice, signs of immunosenescence were clearly detectable in old mice as evidenced by decreased hematopoietic cell numbers in the thymus, spleen, and lymph nodes. In line with earlier studies [8, 56], the compartment of Th cells in the lymph nodes was reduced in old mice, but showed an enhanced inflammatory potential reflected by an increased production of proinflammatory cytokines upon polyclonal stimulation. This was accompanied by an increased antigen-specific reactivity towards G6PI upon immunization in old versus young animals. That reflects the increased severity of symptoms observed in elder onset RA compared to young onset RA patients [55, 57]. Naive T cells in the aged lymph nodes are insufficiently maintained due to a lacking access to IL-7 as consequence of structural changes in the tissue [8]. However, these maintenance problems are not evident in the spleen [8]. In line with this, we did observe a strong reduction in conventional Th cells (Tcons) in the peripheral LNs, but not in the spleen. The unchanged effector/memory phenotype of the Th cells suggested a decrease of naive Th cells in aged lymph nodes, but not of functional effector Th cells. An alteration in the structural organization of LNs in aged mice results in a hampered migration of T cells and APCs [6, 7] and thereby potentially delayed the induction of arthritis upon immunization. Comparable delays in the initiation of an immune response in aged mice have been observed in mouse models for EAE [58], DTH [7], and viral clearance [7, 59].

In contrast to the Tcon compartment, the absolute Treg number was not altered and thus resulted in an increased Treg/Tcon ratio among the generally reduced Th cell compartment. Together with non-altered suppressive capacities, this likely outbalances the increased frequencies of auto-reactive Th cells in old mice. Although many reports presented data on the fate of the overall T effector/memory compartment, it remains completely unresolved, how for example the Treg-Th17 axis is balanced in age [56]. Taking in consideration all aforementioned findings, the observed delayed onset of arthritis in old mice might be the consequence of altered structure of lymphoid organs and thus, in spatial insufficiency for priming events [5–8].

Alongside the alteration in the T cell compartment, we detected the increased number of neutrophils in the joint and altered capacities of myeloid cells to produce proinflammatory mediators, i.e., COX-derived PGE_2_ and TXB_2_ in aged animals. In contrast to the enhanced ratio of autoreactive TNFα-producing Th cells upon inflammatory arthritic response, the cellularity and production of proinflammatory mediators was restored to levels found in young mice.

The stromal compartment has been shown to sustain or even exaggerate the pathological signs of RA [23, 24]. We did not observe any major differences in the population of CD90^+^CD45^-^ FLS, which have been associated with perpetuating chronic inflammation in RA driven by increased synovial tissue vascularization [23–25, 60]. While the population of the CD90^-^CD45^-^ FLS was reduced in the joints of old mice in steady state, their numbers were restored upon arthritis. As the CD90^+^CD45^-^ FLS differentiate from CD90^-^CD45^-^ FLS [25], reduced initial numbers might delay the onset of an arthritic response. The restoration of the CD90^-^CD45^-^ FLS numbers upon arthritis points to a still sufficient hyperplastic capacity of old FLS to drive an inflammatory arthritis response. Such an increased hyperplasia to restore their numbers is supported by the overall increased glycolytic activity of the old FLS and by their slightly increased β-galactosidase activity upon arthritis, both signs for fibroblast activation in the background of arthritis [37, 39]. However, we did not observe an increased aggressiveness of these hyperplastic FLS in an invasion assay, which is in line with a pathologically proinflammatory potential within the CD90^+^CD45^-^ FLS, but not within CD90^-^CD45^-^ FLS [23, 24]. However, these results should be interpreted carefully, because recently Wei et al. showed that already upon 2 passages of cell culture, FLS might lose their positional identity associated with disease progression and converge to comparable signature gene expressions [25]. In addition to this, a more in-depth analysis of FAPα co-expression [24] or CD34 co-expression [23] among FLS is needed to evaluate subset functionality among FLS from young and old mice.

Apart from expected signs of persistent systemic low-grade inflammation in steady state, we could not detect any functional changes in cells derived from old mice compared to cells from young mice upon challenge that is ultimately illustrated by an unaltered arthritis response in the different mouse strains at young and old ages. All mice investigated in the presented study were not only inbred but also were maintained under SPF conditions. To limit any alteration in the microbiome of old mice, which eventually accumulates species modulating the onset and development of arthritis [61–64], all mice had a shared source of food.

## Conclusions

In the presented work, we did not find any significant increase in the severity of G6PI-induced arthritis in old compared to young mice. However, all old mice clearly developed age-related changes in the immune system reflecting inflammaging and immunosenescence in old mice compared to their young counterparts. In contrast to an expected higher susceptibility for arthritis of old mice, we observed a delayed onset of G6PI-induced arthritis when compared to young mice. With the G6PI-induced arthritis model being highly synchronized in onset and progression, and being applicable to DBA/1 and B6.NQ mouse strains, we excluded a mouse strain-specific abnormality of the age relationship of G6PI-induced arthritis. Thus, we demonstrated that overall incidence and severity of experimental arthritis is not age-related. We here propose a model in that arthritis synchronously develops and progresses age-independent under SPF conditions, which is of high value to study potential factors connected with the incidence of arthritis during lifetime.

## Supplementary Information

**Additional file 1: Supplement-Figure 1.** Sex-specifically delayed onset of G6PI-induced Arthritis in DBA/1 mice. DBA/1 mice analyzed in Figure 1 in a G6PI-induced arthritis were separated into young female (n=38), young male cohorts (n=36), old female (n=15), and old male cohorts (n=11). **a)** Comparable with Figure 1, the scores were summarized according to sex- and age-discriminated cohorts in the diagram. **b, c)** Scores at day 9 (b) and day 14 (c) are shown separately. **d)** Young (n=20) and old (n=11) female B6.NQ mice from the cohort shown in Figure 1 are separately shown in the diagram. **e)** Scores of the day 9 intervals of the female B6.NQ mice are separately shown. Statistical testing was performed as described in the methods section.

**Additional file 2: Supplement-Figure 2.** Comparable recovery of young and old mice upon G6PI-induced arthritis. Young (n=3) or old (n=3) DBA/1 mice were immunized with G6PI. Upon recovery from arthritis (arthritis >d56), histology was performed as described by Lories and colleagues [65]. Paws from non-immunized young (n=3) and old (n=3) DBA/1 mice were used as controls (naïve). Representative sections stained with H&E are shown. Bars indicate size of the area (200 μm). **a)** Growth plate structure is shown. **b)** Synovial space with attached synovial membrane is shown.

**Additional file 3: Supplement-Figure 3.** Old mice have reduced peritoneal myeloid cells. Peritoneal lavage from young (average age of 9 weeks, n=5) or old (average age of 113 weeks) mice was stained with antibodies against F4/80, CD11b, CD19, CD90 and CD45 and analyze by flow cytometry. **a)** Gating strategy. **b)** Distribution of subsets among living CD45+ is shown in the pie charts: myeloid cells = CD45^+^ and F4/80^+^ or CD11b^+^, T cells: CD45^+^F4/80^-^CD11b^-^CD90^+^; B cells: CD45^+^F4/80^-^CD11b^-^CD90^-^CD19^+^. Statistical testing was performed as described in the methods section.

**Additional file 4: Supplement-Figure 4.** Old mice overall possess less FoxP3^-^ Th cells, but more Helios^+^ FoxP3^-^ Th cells. **a-f**) Spleens, thymi or peripheral LN (inguinal, brachial, axillary) cells from young (average age of 18 weeks) and old (average age of 101 week) mice were collected. **a)** Total CD45^+^ cell counts are summarized (n=8/ group). **b, c)** Thymocytes (n=8 mice/ age group) were stained intracellularly for FoxP3, CD4, CD8 and CD45. CD4^+^ or CD8^+^ single-positive cells among CD45^+^ thymocytes were analyzed as represented in the FACS plots and summarized in the diagrams (b). Frequencies of FoxP3^+^ or FoxP3^-^CD4^+^ cells among CD45^+^ thymocytes are summarized in (c). **d, e)** Splenocytes (d) and peripheral LN cells (e) were stained as described in (c). Data are summarized in the box plots (n=8/ group). **f)** Peripheral LN cells were intracellularly stained for CD45, CD4, FoxP3, RORγt, GATA3 (all n=8) and Helios (n=5). Frequencies of the indicated FoxP3^-^CD4^+^ populations among CD45^+^ cells are shown. Statistical testing was performed as described in the methods section.

**Additional file 5: Supplement-Tables.** Ages of analyzed mice. Every age of any mouse analyzed is shown in the tables.

## Data Availability

The datasets during and/or analyzed during the current study available from the corresponding author on reasonable request.

## Abbreviations

APCs: Antigen-presenting cells
CFA: Complete Freund’s Adjuvant
COX: Cyclooxygenase
ECAR: Extra Cellular Acidification Rate
FLS: Fibroblast-like synoviocytes
G6PI: Glucose-6-phosphate isomerase
LM: Lipid mediators
LN: Lymph nodes
LOX: Lipoxygenase
OCR: Oxygen consumption rate
RA: Rheumatoid arthritis
SACM: *Staphylococcus (S.) aureus* conditioned medium
SASP: Senescence-associated secretory phenotype
SP: Spleen
SPM: Specialized proresolving mediators
Tcon: Conventional T cells
TCR: T cell receptor
Th: T helper
Thy: Thymus
Treg: Regulatory T cell

## References

[1] Franceschi C, Garagnani P, Parini P, Giuliani C, Santoro A (2018). Inflammaging: a new immune-metabolic viewpoint for age-related diseases. Nat Rev Endocrinol.

[2] Fulop T, Larbi A, Dupuis G, Le Page A, Frost EH, Cohen AA (2017). Immunosenescence and inflamm-aging as two sides of the same coin: friends or foes?. Front Immunol.

[3] Ray D, Yung R (2018). Immune senescence, epigenetics and autoimmunity. Clin Immunol.

[4] Nikolich-Zugich J (2018). The twilight of immunity: emerging concepts in aging of the immune system. Nat Immunol.

[5] Thompson HL, Smithey MJ, Uhrlaub JL, Jeftic I, Jergovic M, White SE (2019). Lymph nodes as barriers to T-cell rejuvenation in aging mice and nonhuman primates. Aging Cell.

[6] Shaw AC, Goldstein DR, Montgomery RR (2013). Age-dependent dysregulation of innate immunity. Nat Rev Immunol.

[7] Richner JM, Gmyrek GB, Govero J, Tu Y, van der Windt GJ, Metcalf TU (2015). Age-dependent cell trafficking defects in draining lymph nodes impair adaptive immunity and control of West Nile virus infection. PLoS Pathog.

[8] Becklund BR, Purton JF, Ramsey C, Favre S, Vogt TK, Martin CE, Spasova DS, Sarkisyan G, LeRoy E, Tan JT, Wahlus H, Bondi-Boyd B, Luther SA, Surh CD (2016). The aged lymphoid tissue environment fails to support naive T cell homeostasis. Sci Rep.

[9] Franceschi C, Garagnani P, Vitale G, Capri M, Salvioli S (2017). Inflammaging and 'Garb-aging'. Trends Endocrinol Metab.

[10] De Maeyer RPH, van de Merwe RC, Louie R, Bracken OV, Devine OP, Goldstein DR (2020). Blocking elevated p38 MAPK restores efferocytosis and inflammatory resolution in the elderly. Nat Immunol.

[11] Campisi J, Kapahi P, Lithgow GJ, Melov S, Newman JC, Verdin E (2019). From discoveries in ageing research to therapeutics for healthy ageing. Nature..

[12] Crowson CS, Matteson EL, Myasoedova E, Michet CJ, Ernste FC, Warrington KJ, Davis JM, Hunder GG, Therneau TM, Gabriel SE (2011). The lifetime risk of adult-onset rheumatoid arthritis and other inflammatory autoimmune rheumatic diseases. Arthritis Rheum.

[13] Kronzer VL, Davis JM (2021). Etiologies of rheumatoid arthritis: update on mucosal, genetic, and cellular pathogenesis. Curr Rheumatol Rep.

[14] Rees F, Doherty M, Grainge MJ, Lanyon P, Zhang W (2017). The worldwide incidence and prevalence of systemic lupus erythematosus: a systematic review of epidemiological studies. Rheumatology (Oxford).

[15] Park JS, Hong JY, Park YS, Han K, Suh SW (2018). Trends in the prevalence and incidence of ankylosing spondylitis in South Korea, 2010-2015 and estimated differences according to income status. Sci Rep.

[16] Brekke LK, Diamantopoulos AP, Fevang BT, Abetamus J, Espero E, Gjesdal CG (2017). Incidence of giant cell arteritis in Western Norway 1972-2012: a retrospective cohort study. Arthritis Res Ther.

[17] Myasoedova E, Davis J, Matteson EL, Crowson CS. Is the epidemiology of rheumatoid arthritis changing? Results from a population-based incidence study, 1985-2014. Ann Rheum Dis 2020;79(4):440-4. 10.1136/annrheumdis-2019-216694. 10.1136/annrheumdis-2019-216694 PMC7085464 32066556

[18] Doran MF, Pond GR, Crowson CS, O'Fallon WM, Gabriel SE (2002). Trends in incidence and mortality in rheumatoid arthritis in Rochester, Minnesota, over a forty-year period. Arthritis Rheum.

[19] Humphreys JH, Verstappen SM, Hyrich KL, Chipping JR, Marshall T, Symmons DP (2013). The incidence of rheumatoid arthritis in the UK: comparisons using the 2010 ACR/EULAR classification criteria and the 1987 ACR classification criteria. Results from the Norfolk Arthritis Register. Ann Rheum Dis.

[20] Ito H, Ogura T, Hirata A, Takenaka S, Mizushina K, Fujisawa Y, Katagiri T, Hayashi N, Kameda H (2017). Global assessments of disease activity are age-dependent determinant factors of clinical remission in rheumatoid arthritis. Semin Arthritis Rheum.

[21] Bukhari M, Lunt M, Barton A, Bunn D, Silman A, Symmons D (2007). Increasing age at symptom onset is associated with worse radiological damage at presentation in patients with early inflammatory polyarthritis. Ann Rheum Dis.

[22] Kiener HP, Watts GF, Cui Y, Wright J, Thornhill TS, Skold M (2010). Synovial fibroblasts self-direct multicellular lining architecture and synthetic function in three-dimensional organ culture. Arthritis Rheum.

[23] Mizoguchi F, Slowikowski K, Wei K, Marshall JL, Rao DA, Chang SK, Nguyen HN, Noss EH, Turner JD, Earp BE, Blazar PE, Wright J, Simmons BP, Donlin LT, Kalliolias GD, Goodman SM, Bykerk VP, Ivashkiv LB, Lederer JA, Hacohen N, Nigrovic PA, Filer A, Buckley CD, Raychaudhuri S, Brenner MB (2018). Functionally distinct disease-associated fibroblast subsets in rheumatoid arthritis. Nat Commun.

[24] Croft AP, Campos J, Jansen K, Turner JD, Marshall J, Attar M, Savary L, Wehmeyer C, Naylor AJ, Kemble S, Begum J, Dürholz K, Perlman H, Barone F, McGettrick HM, Fearon DT, Wei K, Raychaudhuri S, Korsunsky I, Brenner MB, Coles M, Sansom SN, Filer A, Buckley CD (2019). Distinct fibroblast subsets drive inflammation and damage in arthritis. Nature..

[25] Wei K, Korsunsky I, Marshall JL, Gao A, Watts GFM, Major T (2020). Notch signalling drives synovial fibroblast identity and arthritis pathology. Nature..

[26] Tarjanyi O, Boldizsar F, Nemeth P, Mikecz K, Glant TT (2009). Age-related changes in arthritis susceptibility and severity in a murine model of rheumatoid arthritis. Immun Ageing.

[27] Schubert D, Maier B, Morawietz L, Krenn V, Kamradt T (2004). Immunization with glucose-6-phosphate isomerase induces T cell-dependent peripheral polyarthritis in genetically unaltered mice. J Immunol.

[28] Backlund J, Li C, Jansson E, Carlsen S, Merky P, Nandakumar KS (2013). C57BL/6 mice need MHC class II Aq to develop collagen-induced arthritis dependent on autoreactive T cells. Ann Rheum Dis.

[29] Rudolph KL, Chang S, Lee HW, Blasco M, Gottlieb GJ, Greider C, DePinho RA (1999). Longevity, stress response, and cancer in aging telomerase-deficient mice. Cell..

[30] Win SJ, Kuhl AA, Sparwasser T, Hunig T, Kamradt T (2016). In vivo activation of Treg cells with a CD28 superagonist prevents and ameliorates chronic destructive arthritis in mice. Eur J Immunol.

[31] Jordan PM, Gerstmeier J, Pace S, Bilancia R, Rao Z, Borner F (2020). Staphylococcus aureus-derived alpha-hemolysin evokes generation of specialized pro-resolving mediators promoting inflammation resolution. Cell Rep.

[32] Werz O, Gerstmeier J, Libreros S, De la Rosa X, Werner M, Norris PC (2018). Human macrophages differentially produce specific resolvin or leukotriene signals that depend on bacterial pathogenicity. Nat Commun.

[33] Colas RA, Shinohara M, Dalli J, Chiang N, Serhan CN (2014). Identification and signature profiles for pro-resolving and inflammatory lipid mediators in human tissue. Am J Phys Cell Phys.

[34] Werner M, Jordan PM, Romp E, Czapka A, Rao Z, Kretzer C, Koeberle A, Garscha U, Pace S, Claesson HE, Serhan CN, Werz O, Gerstmeier J (2019). Targeting biosynthetic networks of the proinflammatory and proresolving lipid metabolome. FASEB J.

[35] Locksley RM, Heinzel FP, Sadick MD, Holaday BJ, Gardner KD (1987). Murine cutaneous leishmaniasis: susceptibility correlates with differential expansion of helper T-cell subsets. Ann Inst Pasteur Immunol.

[36] Fearon U, Hanlon MM, Wade SM, Fletcher JM (2019). Altered metabolic pathways regulate synovial inflammation in rheumatoid arthritis. Clin Exp Immunol.

[37] de Oliveira PG, Farinon M, Sanchez-Lopez E, Miyamoto S, Guma M (2019). Fibroblast-like synoviocytes glucose metabolism as a therapeutic target in rheumatoid arthritis. Front Immunol.

[38] Dimri GP, Lee X, Basile G, Acosta M, Scott G, Roskelley C, Medrano EE, Linskens M, Rubelj I, Pereira-Smith O (1995). A biomarker that identifies senescent human cells in culture and in aging skin in vivo. Proc Natl Acad Sci U S A.

[39] Maier AB, Westendorp RG (2007). D VANH. Beta-galactosidase activity as a biomarker of replicative senescence during the course of human fibroblast cultures. Ann N Y Acad Sci.

[40] Serhan CN (2014). Pro-resolving lipid mediators are leads for resolution physiology. Nature..

[41] Uderhardt S, Herrmann M, Oskolkova OV, Aschermann S, Bicker W, Ipseiz N, Sarter K, Frey B, Rothe T, Voll R, Nimmerjahn F, Bochkov VN, Schett G, Krönke G (2012). 12/15-lipoxygenase orchestrates the clearance of apoptotic cells and maintains immunologic tolerance. Immunity..

[42] Culemann S, Gruneboom A, Nicolas-Avila JA, Weidner D, Lammle KF, Rothe T (2019). Locally renewing resident synovial macrophages provide a protective barrier for the joint. Nature..

[43] Jenkins SJ, Ruckerl D, Cook PC, Jones LH, Finkelman FD, van Rooijen N, MacDonald AS, Allen JE (2011). Local macrophage proliferation, rather than recruitment from the blood, is a signature of TH2 inflammation. Science..

[44] Linehan E, Dombrowski Y, Snoddy R, Fallon PG, Kissenpfennig A, Fitzgerald DC (2014). Aging impairs peritoneal but not bone marrow-derived macrophage phagocytosis. Aging Cell.

[45] Thornton AM, Lu J, Korty PE, Kim YC, Martens C, Sun PD, Shevach EM (2019). Helios(+) and Helios(-) Treg subpopulations are phenotypically and functionally distinct and express dissimilar TCR repertoires. Eur J Immunol.

[46] Ross EM, Bourges D, Hogan TV, Gleeson PA, van Driel IR (2014). Helios defines T cells being driven to tolerance in the periphery and thymus. Eur J Immunol.

[47] Frey O, Reichel A, Bonhagen K, Morawietz L, Rauchhaus U, Kamradt T (2010). Regulatory T cells control the transition from acute into chronic inflammation in glucose-6-phosphate isomerase-induced arthritis. Ann Rheum Dis.

[48] Sakaguchi N, Takahashi T, Hata H, Nomura T, Tagami T, Yamazaki S, Sakihama T, Matsutani T, Negishi I, Nakatsuru S, Sakaguchi S (2003). Altered thymic T-cell selection due to a mutation of the ZAP-70 gene causes autoimmune arthritis in mice. Nature..

[49] Keffer J, Probert L, Cazlaris H, Georgopoulos S, Kaslaris E, Kioussis D, Kollias G (1991). Transgenic mice expressing human tumour necrosis factor: a predictive genetic model of arthritis. EMBO J.

[50] Stuart JM, Dixon FJ (1983). Serum transfer of collagen-induced arthritis in mice. J Exp Med.

[51] Korganow AS, Ji H, Mangialaio S, Duchatelle V, Pelanda R, Martin T, Degott C, Kikutani H, Rajewsky K, Pasquali JL, Benoist C, Mathis D (1999). From systemic T cell self-reactivity to organ-specific autoimmune disease via immunoglobulins. Immunity..

[52] Weyand CM, Yang Z, Goronzy JJ (2014). T-cell aging in rheumatoid arthritis. Curr Opin Rheumatol.

[53] Asquith DL, Miller AM, McInnes IB, Liew FY (2009). Animal models of rheumatoid arthritis. Eur J Immunol.

[54] Caplazi P, Baca M, Barck K, Carano RA, DeVoss J, Lee WP (2015). Mouse models of rheumatoid arthritis. Vet Pathol.

[55] Nilsson J, Andersson MLE, Hafstrom I, Svensson B, Forslind K, Ajeganova S (2021). Influence of age and sex on disease course and treatment in rheumatoid arthritis. Open Access Rheumatol.

[56] Nikolich-Zugich J (2014). Aging of the T cell compartment in mice and humans: from no naive expectations to foggy memories. J Immunol.

[57] Ke Y, Dai X, Xu D, Liang J, Yu Y, Cao H, Chen W, Lin J (2021). Features and outcomes of elderly rheumatoid arthritis: does the age of onset matter? A Comparative Study From a Single Center in China. Rheumatol Ther.

[58] Matejuk A, Hopke C, Vandenbark AA, Hurn PD, Offner H (2005). Middle-age male mice have increased severity of experimental autoimmune encephalomyelitis and are unresponsive to testosterone therapy. J Immunol.

[59] Toapanta FR, Ross TM (2009). Impaired immune responses in the lungs of aged mice following influenza infection. Respir Res.

[60] Zhang F, Wei K, Slowikowski K, Fonseka CY, Rao DA, Kelly S (2019). Defining inflammatory cell states in rheumatoid arthritis joint synovial tissues by integrating single-cell transcriptomics and mass cytometry. Nat Immunol.

[61] Abdollahi-Roodsaz S, Joosten LA, Koenders MI, Devesa I, Roelofs MF, Radstake TR (2008). Stimulation of TLR2 and TLR4 differentially skews the balance of T cells in a mouse model of arthritis. J Clin Invest.

[62] Liu X, Zeng B, Zhang J, Li W, Mou F, Wang H, Zou Q, Zhong B, Wu L, Wei H, Fang Y (2016). Role of the Gut Microbiome in Modulating Arthritis Progression in Mice. Sci Rep.

[63] Teng F, Felix KM, Bradley CP, Naskar D, Ma H, Raslan WA, Wu HJJ (2017). The impact of age and gut microbiota on Th17 and Tfh cells in K/BxN autoimmune arthritis. Arthritis Res Ther.

[64] Scher JU, Littman DR, Abramson SB (2016). Microbiome in inflammatory arthritis and human rheumatic diseases. Arthritis Rheum.

[65] Lories RJ, Matthys P, de Vlam K, Derese I, Luyten FP (2004). Ankylosing enthesitis, dactylitis, and onychoperiostitis in male DBA/1 mice: a model of psoriatic arthritis. Ann Rheum Dis.

