# Supplementary figures and images for: Incidence and severity of G6PI-induced arthritis are not increased in genetically distinct mouse strains upon aging
Source: Arthritis Res Ther. 2021 Aug 24;23:222. doi: 10.1186/s13075-021-02596-7 (PMC8383389; doi:10.1186/s13075-021-02596-7)

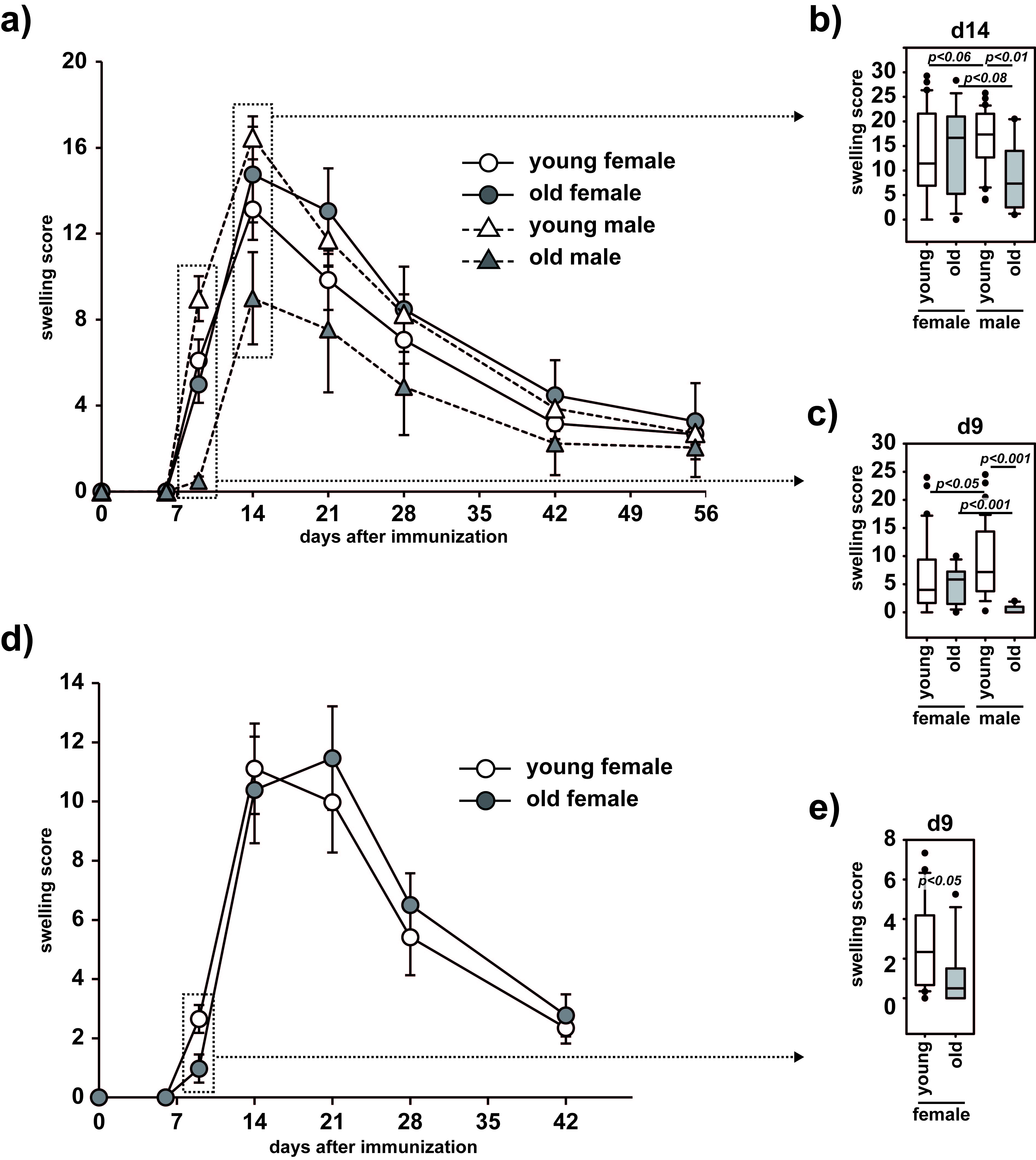

Supplement: Supplementary file 1 — Additional file 1: Supplement-Figure 1. Sex-specifically delayed onset of G6PI-induced Arthritis in DBA/1 mice. DBA/1 mice analyzed in Figure 1 in a G6PI-induced arthritis were separated into young female (n=38), young male cohorts (n=36), old female (n=15), and old male cohorts (n=11). a) Comparable with Figure 1, the scores were summarized according to sex- and age-discriminated cohorts in the diagram. b, c) Scores at day 9 (b) and day 14 (c) are shown separately. d) Young (n=20) and old (n=11) female B6.NQ mice from the cohort shown in Figure 1 are separately shown in the diagram. e) Scores of the day 9 intervals of the female B6.NQ mice are separately shown. Statistical testing was performed as described in the methods section. [file 13075_2021_2596_MOESM1_ESM.jpg]

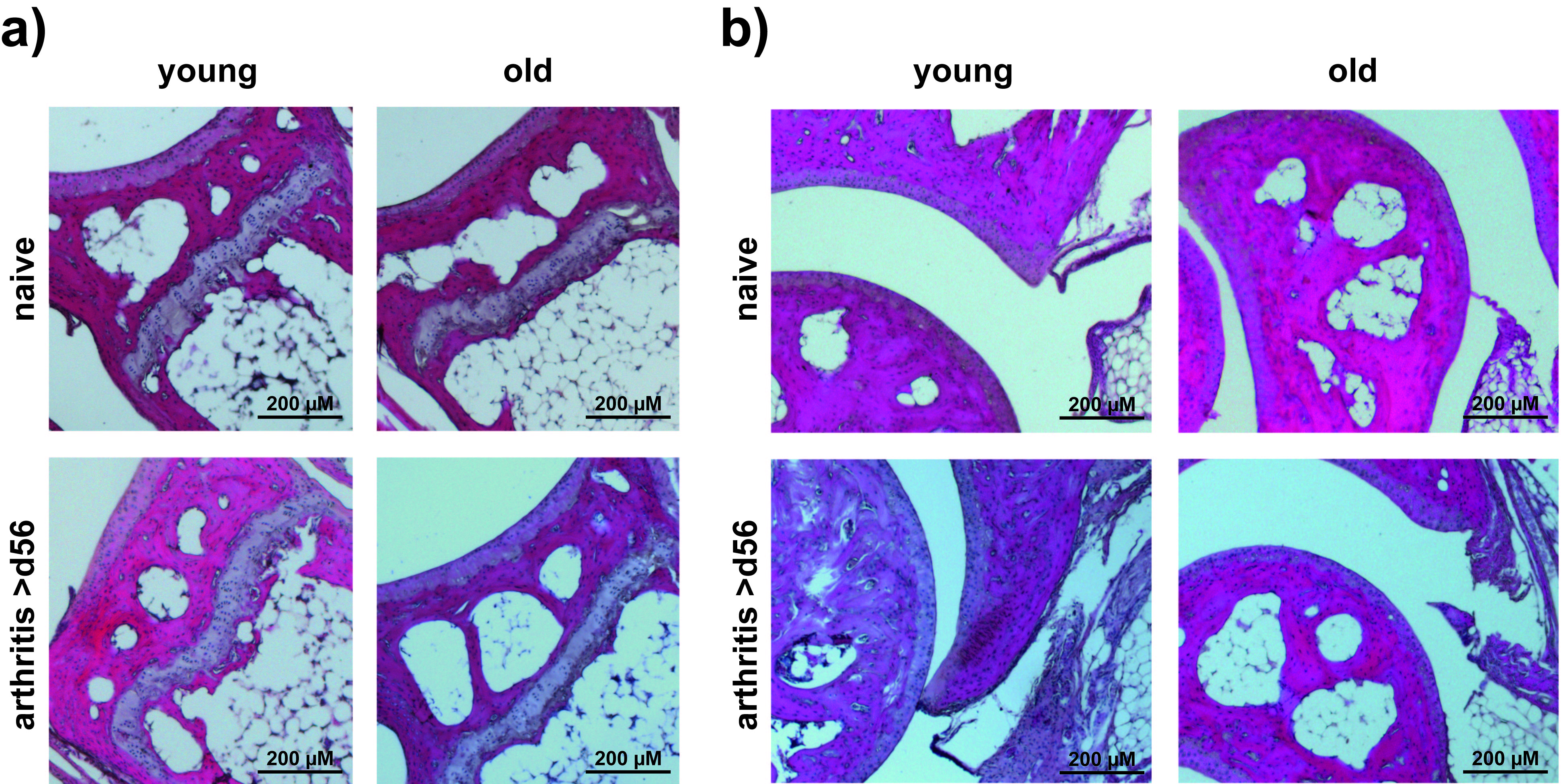

Supplement: Supplementary file 2 — Additional file 2: Supplement-Figure 2. Comparable recovery of young and old mice upon G6PI-induced arthritis. Young (n=3) or old (n=3) DBA/1 mice were immunized with G6PI. Upon recovery from arthritis (arthritis >d56), histology was performed as described by Lories and colleagues [65]. Paws from non-immunized young (n=3) and old (n=3) DBA/1 mice were used as controls (naïve). Representative sections stained with H&E are shown. Bars indicate size of the area (200 μm). a) Growth plate structure is shown. b) Synovial space with attached synovial membrane is shown. [file 13075_2021_2596_MOESM2_ESM.jpg]

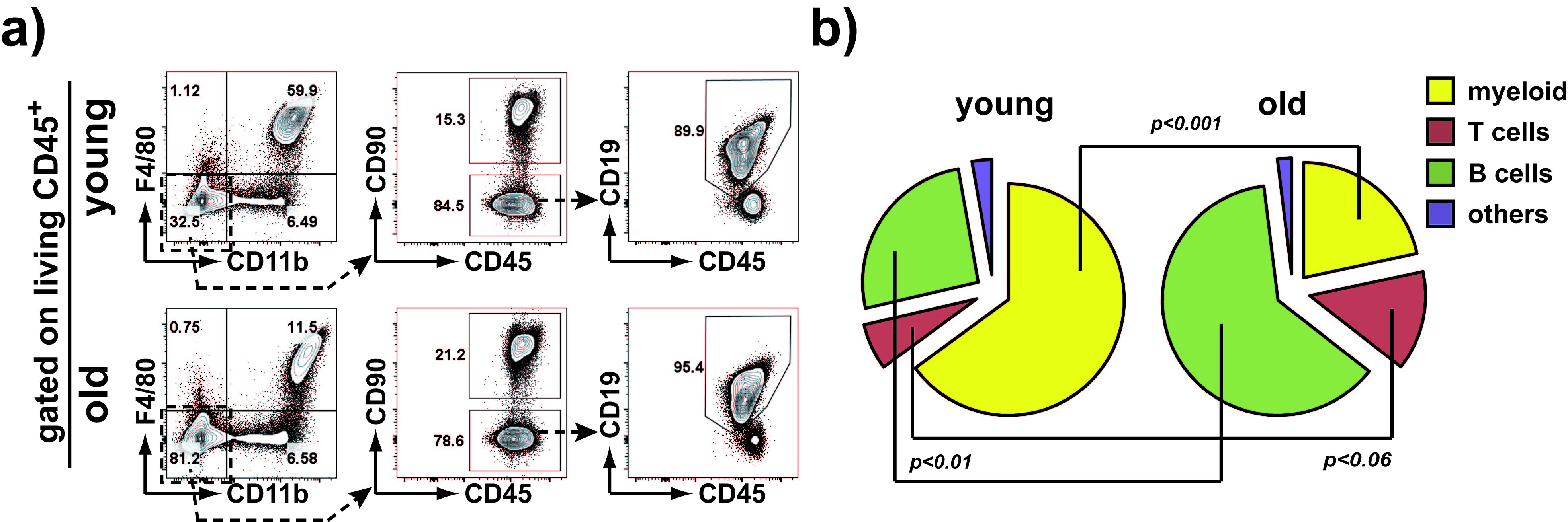

Supplement: Supplementary file 3 — Additional file 3: Supplement-Figure 3. Old mice have reduced peritoneal myeloid cells. Peritoneal lavage from young (average age of 9 weeks, n=5) or old (average age of 113 weeks) mice was stained with antibodies against F4/80, CD11b, CD19, CD90 and CD45 and analyze by flow cytometry. a) Gating strategy. b) Distribution of subsets among living CD45+ is shown in the pie charts: myeloid cells = CD45+ and F4/80+ or CD11b+, T cells: CD45+F4/80-CD11b-CD90+; B cells: CD45+F4/80-CD11b-CD90-CD19+. Statistical testing was performed as described in the methods section. [file 13075_2021_2596_MOESM3_ESM.jpg]

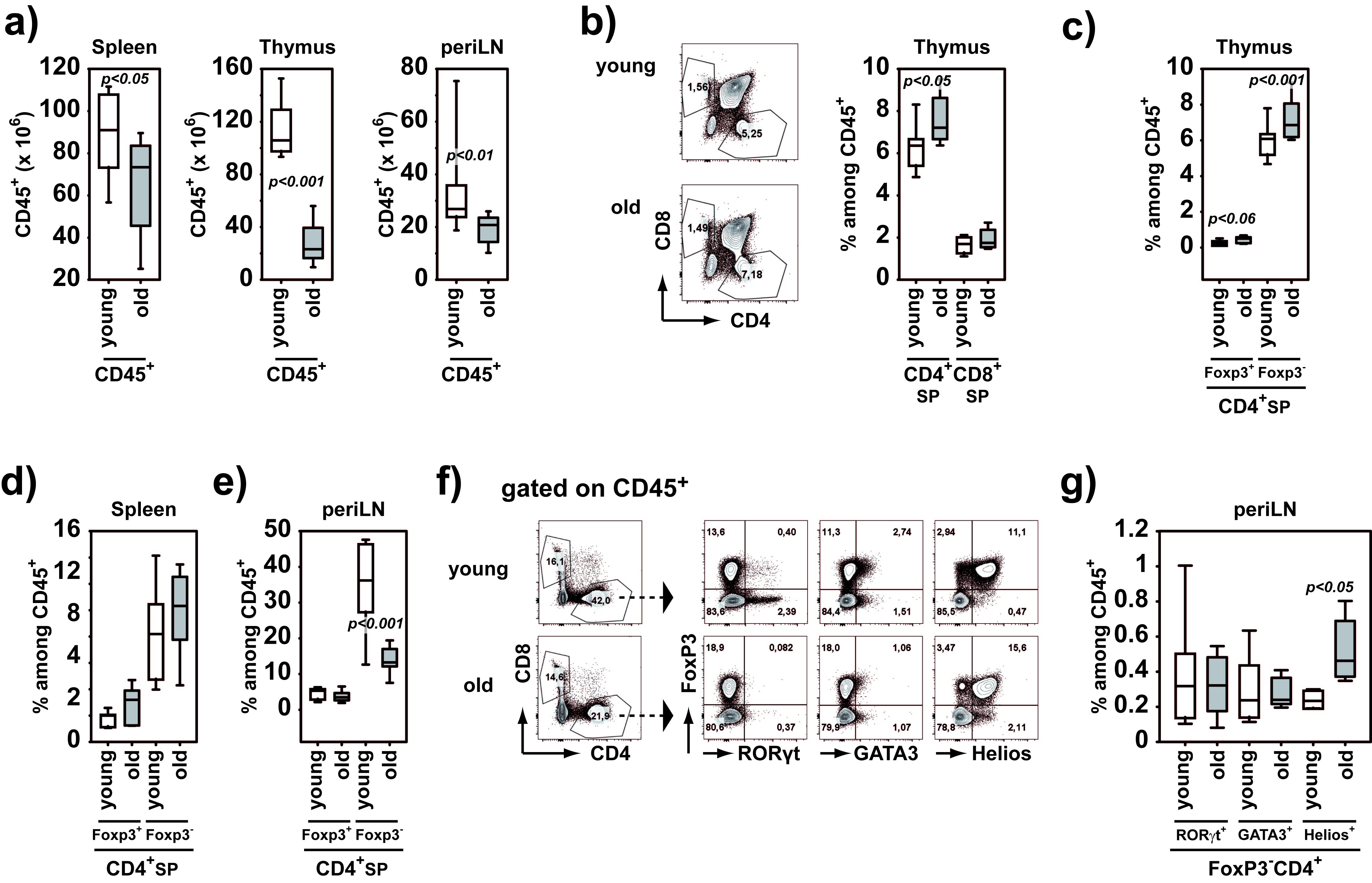

Supplement: Supplementary file 4 — Additional file 4: Supplement-Figure 4. Old mice overall possess less FoxP3- Th cells, but more Helios+ FoxP3- Th cells. a-f) Spleens, thymi or peripheral LN (inguinal, brachial, axillary) cells from young (average age of 18 weeks) and old (average age of 101 week) mice were collected. a) Total CD45+ cell counts are summarized (n=8/ group). b, c) Thymocytes (n=8 mice/ age group) were stained intracellularly for FoxP3, CD4, CD8 and CD45. CD4+ or CD8+ single-positive cells among CD45+ thymocytes were analyzed as represented in the FACS plots and summarized in the diagrams (b). Frequencies of FoxP3+ or FoxP3-CD4+ cells among CD45+ thymocytes are summarized in (c). d, e) Splenocytes (d) and peripheral LN cells (e) were stained as described in (c). Data are summarized in the box plots (n=8/ group). f) Peripheral LN cells were intracellularly stained for CD45, CD4, FoxP3, RORγt, GATA3 (all n=8) and Helios (n=5). Frequencies of the indicated FoxP3-CD4+ populations among CD45+ cells are shown. Statistical testing was performed as described in the methods section. [file 13075_2021_2596_MOESM4_ESM.jpg]

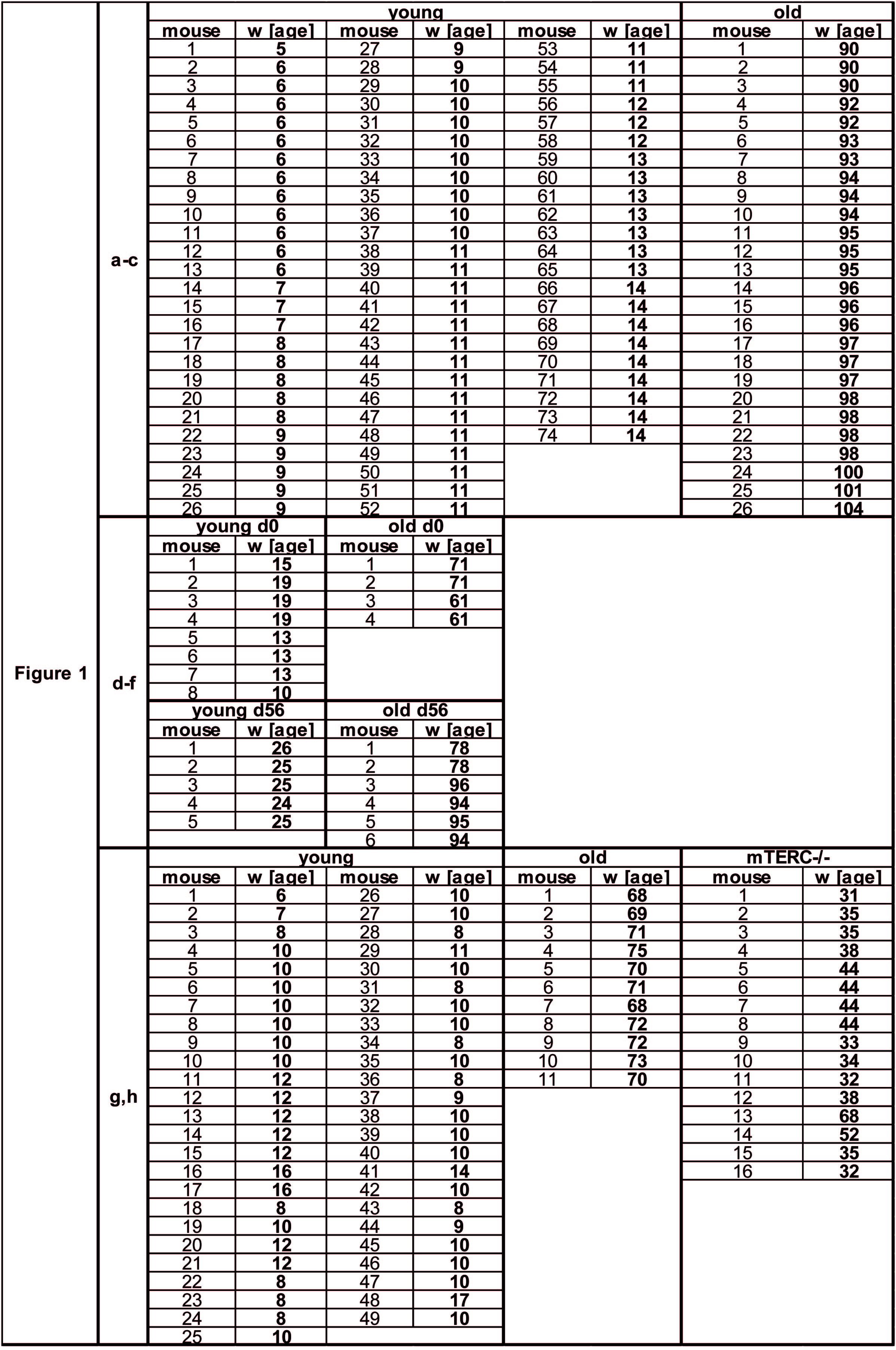

Supplement: Supplementary file 5 — Additional file 5: Supplement-Tables. Ages of analyzed mice. Every age of any mouse analyzed is shown in the tables. [file 13075_2021_2596_MOESM5_ESM.zip › Andreas et al - revised Table for Fig 1.jpg]

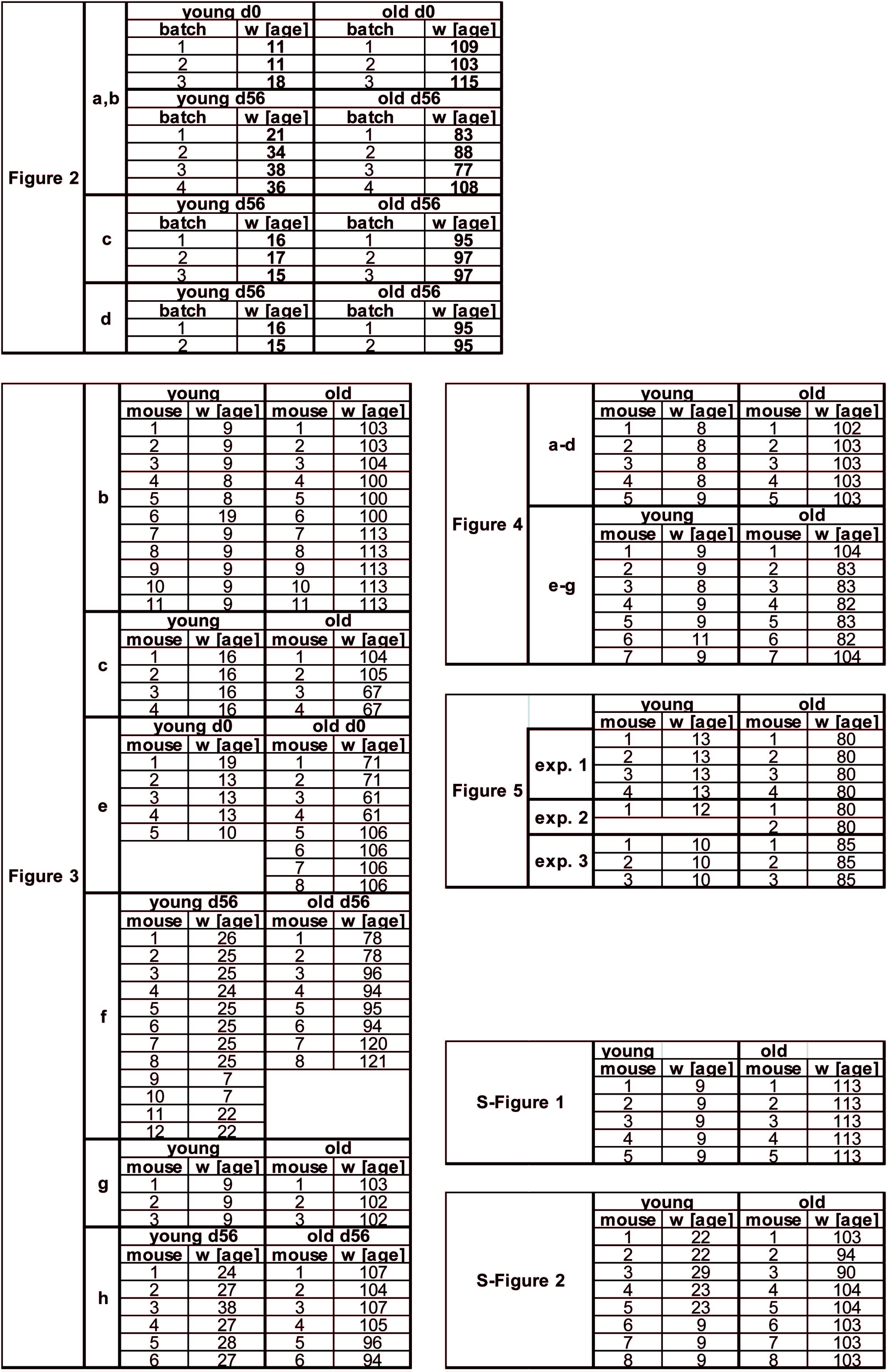

Supplement: Supplementary file 5 — Additional file 5: Supplement-Tables. Ages of analyzed mice. Every age of any mouse analyzed is shown in the tables. [file 13075_2021_2596_MOESM5_ESM.zip › Andreas et al - revised Table for Fig 2-5.jpg]
